# Ginsenoside-Rb1 for Ischemic Stroke: A Systematic Review and Meta-analysis of Preclinical Evidence and Possible Mechanisms

**DOI:** 10.3389/fphar.2020.00285

**Published:** 2020-03-31

**Authors:** Yi-Hua Shi, Yan Li, Yong Wang, Zhen Xu, Huan Fu, Guo-Qing Zheng

**Affiliations:** Department of Neurology, The Second Affiliated Hospital and Yuying Children’s Hospital of Wenzhou Medical University, Wenzhou, China

**Keywords:** animal model, ischemic stroke, Ginsenoside-Rb1, Ginseng, neuroprotective mechanisms

## Abstract

**Background:**

Ischemic stroke is the most common type of stroke, while pharmacological therapy options are limited. Ginsenosides are the major bioactive compounds in Ginseng and have been found to have various pharmacological effects in the nervous system. In the present study, we sought to evaluate the effects of Ginsenoside-Rb1 (G-Rb1), an important ingredient of ginsenosides, and the probable neuroprotective mechanisms in experimental ischemic strokes.

**Methods:**

Studies of G-Rb1 on ischemic stroke animal models were identified from 7 databases. No clinical trials were included in the analysis. The primary outcome measures were neurological function scores, infarct volume, evans blue content and/or brain water content (BWC). The second outcome measures were the possible neuroprotective mechanisms. All the data were analyzed by Rev Man 5.3.

**Result:**

Pooled preclinical data showed that compared with the controls, G-Rb1 could improve neurological function (Zea Longa (n = 367, P < 0.01); mNSS (n = 70, P < 0.01); Water maze test (n = 48, P < 0.01); Bederson (n = 16, P < 0.01)), infarct area (TTC (n = 211, P < 0.01); HE (n = 26, P < 0.01)), as well as blood-brain barrier function (BWC (n = 64, P < 0.01); Evans blue content (n=26, P < 0.05)). It also can increase BDNF (n = 26, P < 0.01), Gap-43 (n = 16, P < 0.01), SOD (n = 30, P < 0.01), GSH (n = 16, P < 0.01), Nissl-positive cells (n = 12, P < 0.01), Nestin-positive cells (n = 10, P < 0.05), and reduce Caspase-3 (n = 36, P < 0.01), IL-1 (n = 32, P < 0.01), TNF-α (n = 72, P < 0.01), MDA (n = 18, P < 0.01), NO (n = 44, P < 0.01), NOX (n = 32, P < 0.05), ROS (n = 6, P < 0.05), NF-κB (P < 0.05) and TUNEL-positive cells (n = 52, P < 0.01).

**Conclusion:**

Available findings demonstrated the preclinical evidence that G-Rb1 has a potential neuroprotective effect, largely through attenuating brain water content, promoting the bioactivities of neurogenesis, anti-apoptosis, anti-oxidative, anti-inflammatory, energy supplement and cerebral circulation.

## Highlights

- The preclinical systematic review assessed the available evidence of G-Rb1 for experimental ischemic stroke.
- The present study identified 28 preclinical studies involving 937 animals, which largely augments the sample size.
- This study suggests G-Rb1 exerts a neuroprotective function in experiment ischemic stroke.
- The possible mechanisms are anti-apoptosis, anti-oxidation, anti-inflammation, enhancing neurogenesis, energy metabolism, and cerebral circulation.

## Introduction

Globally, stroke is the second most common cause of death and the most common cause of long-term disability (Kassebaum et al., 2016; Feigin et al., 2017), causing a huge burden with approximately 10.3 million new cases of stroke and 113 million disability-adjusted life years every year (Pandian et al., 2018). The two major mechanisms causing brain damage in stroke are ischemic and hemorrhagic, where ischemic stroke occurs more frequently according to the epidemiological research (Meyers et al., 2011; Venketasubramanian et al., 2017).

Ischemic stroke, in most instances, is caused by a transient or permanent occlusion (either by an embolus or by local thrombosis) in cerebral arteries, which leads to a sharp reduction in cerebral blood flow (CBF) (Dirnagl et al., 1999). In order to maintain normal physiological activity, the brain requires substantial amounts of oxygen and glucose as brought by CBF. Immediately after ischemia, neurons are deprived of oxygen and energy, and become unable to preserve normal transmembrane ionic gradients and homoeostasis (Murphy et al., 2008). Cerebral ischemia initiates several pathologic processes, including oxidative and nitrative stress, inflammation, apoptosis, ion imbalance, calcium overload, and energy depletion (Terasaki et al., 2014; Jayaraj et al., 2019), leading to neurovascular unit dysfunction and neurologic deficits in ischemic stroke. In the central area of an ischemic stroke, CBF is severely in deficit and cells die rapidly. But in the ischemic penumbra, an area of damaged but not yet dead brain tissue after focal cerebral ischemia, CBF deficit is milder which makes it a salvageable area (Lo, 2008). Therefore, therapeutic candidates that can prompt restoration of CBF and improve neurological dysfunction have become an important research hotspot.

Intravenously recombinant tissue-type plasminogen activator (tPA) has been approved by the Food and Drug Administration as the effective pharmacological therapy for acute ischemic stroke since 1996 (National Institute of Neurological Disorders and Stroke rt-PA Stroke Study Group, 1995; Sandercock et al., 2012; Berge et al., 2016). However, only a minority of acute ischemic stroke patients can receive it because of the narrow therapeutic time window and several contraindications to its use, especially the risk of a fatal symptomatic intracranial hemorrhage (Demaerschalk et al., 2016). Beyond the beneficial effects, artery recanalization can also lead to severe adverse reactions, for example, cerebral ischemia/reperfusion injuries (Jickling et al., 2014). Given the present dilemma, it is necessary to seek other novel pharmacological treatment modalities.

**Figure 1:**
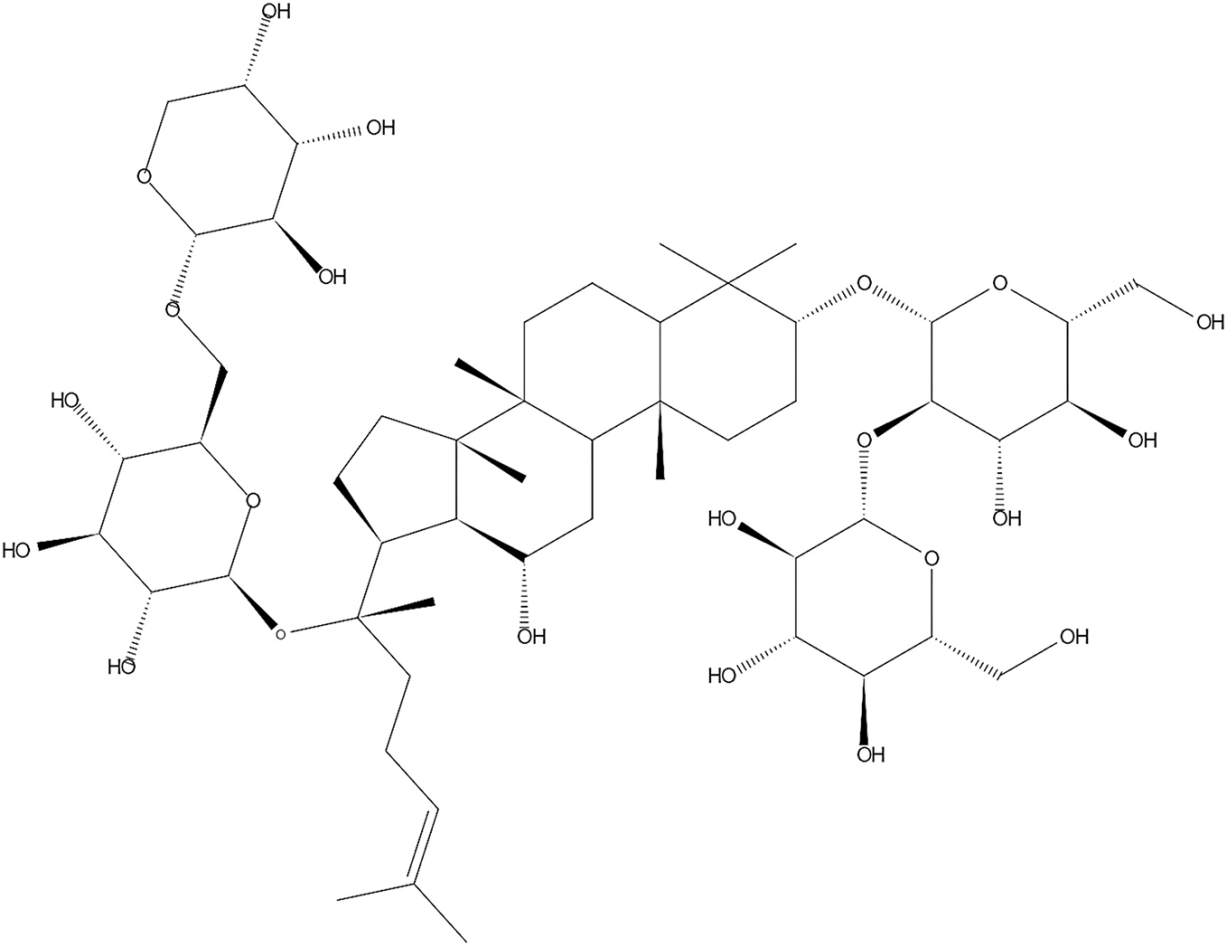
Chemical structures of G-Rb1.

Ginseng is a famous herb medicine and has been used as a tonic to improve a wide variety of disorders for millennia (Lee et al., 2015). In modern time, it is continuously and widely used worldwide (Kim, 2012). Ginsenosides, which are responsible for the pharmacologic effects, are extracted from Ginseng and often divided into two different groups: the 20 (S)-protopanaxatriol group (ginsenosides Re, Rf, Rg1, Rg2, and Rh1) and the 20 (S)- protopanaxadiol group (ginsenosides Ra1, Ra2, Ra3, Rb1, Rb2, Rb3, Rc, Rd, Rg3 and Rh2) (Ong et al., 2015; Jin et al., 2019). Randomized, double-blind, placebo-controlled, multicenter clinical trials have reported that ginsenosides could significantly improve the overall distribution of disability scores on the modified Rankin scale after acute ischemic stroke. It could also improve scores on the National Institutes of Health Stroke Scale with similar mortality and adverse event rates when compared with placebo (Liu et al., 2009; Liu et al., 2012). Ginsenoside-Rb1 (G-Rb1) (the chemical structure is shown in **Figure 1**) is regarded as the major active ingredient (Lee et al., 2015), which has been proven to cause a wide range of biological activities, especially in regards to its neuroprotective role, probably through anti-inflammatory, anti-oxidative, anti-apoptosis, anti-stress and anti-depressive effects *in vivo* and *in vitro* (Kim, 2012; Kim et al., 2013; Ong et al., 2015; Rokot et al., 2016; Wang et al., 2018).

Animal experiments are often conducted to test the effects of a drug before proceeding to clinical trials. However, there are some defects in animal experiments. For one thing, it is not feasible to imitate all aspects of human ischemic stroke in an animal model because of the complex pathophysiology and its heterogeneous nature. For another, most studies are conducted in young animals without any comorbidities, which is different from human stroke as it particularly affects elderly people with the additional risk factor of cerebrovascular diseases (Fluri et al., 2015). Thus, the results of animal experiments are dependent on the type of animal model used (Roberts et al., 2002). The transient or permanent middle cerebral artery occlusion (MCAO) model is one of the closest models to human ischemic stroke, which is characterized by reliable and well-reproducible infarcts and has been applied in the majority of studies on the pathophysiological processes or neuroprotective agents of ischemic stroke (Fluri et al., 2015).

An objective and quantitative systematic review is a type of secondary research, which collates all primary studies that fit prespecified eligibility criteria to solve a specific research problem, which can minimize bias (Higgins et al., 2011). Systematic reviews can offer credible evidence and be helpful for selecting the appropriate drug administration for clinical trials (van Luijk et al., 2013). But up until now, there has been no systematic review to pool the therapeutic effects of G-Rb1 in preclinical studies. Thus, in the present study, we aimed to evaluate the effects and mechanisms of G-Rb1 monotherapy for focal ischemic stroke in animal models using a systematic review approach.

## Materials and Methods

This systematic review and meta-analysis is reported according to the Preferred Reporting Items for Systematic Reviews and Meta-Analyses: The PRISMA Statement (Moher et al., 2010) and our previous study (Zheng et al., 2017).

### Search Strategy

A comprehensive search was performed to find studies evaluating the effects of G-Rb1 treatment on animal models of ischemic stroke from PubMed, Web of Science, the Cochrane Library, Embase, VIP information database, Wanfang Data Information Site, and the Chinese National Knowledge Infrastructure. All of the searches were conducted from the inception to September 27^th^, 2019 without language or publication status restrictions. The following search strategy was used for PubMed and was modified to suit other databases: (ginseng OR ginsenoside OR Ginsenoside-Rb1 OR G-Rb1) AND (stroke OR “middle cerebral artery occlusion” OR “middle carotid artery occlusion” OR MCAO OR “brain infarct*” OR “brain ischemi*” OR “cerebral infarct*” OR “cerebral ischemi*”). The Chinese databases were also searched by the above search terms, translated into Chinese.

### Inclusion Criteria

We included controlled studies evaluating the effects of G-Rb1 treatment on experimental ischemic stroke. To prevent bias, the following inclusion criteria were prespecified: (1) focal cerebral ischemia, induced by transient middle cerebral artery occlusion (MCAO) or permanent MCAO; (2) no restriction on animal species, as well as gender, age, weight and sample size; (3) the experimental group was administered by G-Rb1 monotherapy, while the control group received a vehicle, saline, or positive control drug or no treatment. There was no restriction on dosage, mode and time of initial treatment. The primary outcome measures were neurological function score (NFS), infarct volume (IV), evans blue content and/or brain water content (BWC). The secondary outcome measures were possible mechanisms of G-Rb1 for cerebral ischemic stroke.

### Exclusion Criteria

The prespecified exclusion criteria were: (1) clinical articles, case reports, comments, reviews, abstracts and *in vitro* studies; (2) using global models, traumatic models or hypoxic-ischemic models; (3) not using a cerebral ischemia model; (4) the intervention for the experimental group was a combination of G-Rb1 and another drug; (5) lacking a control group.

### Data Extraction

According to the previous studies (Wang et al., 2017; Cascella et al., 2018), the following details were collected from the included studies independently by two authors: (1) name of the first author and the publication year; (2) the characteristics of animals including animal species, gender, number and weight; (3) type of anesthetic; (4) treatment information, including the establishment of an ischemic stroke model and the method of administration; (5) outcome measures and timings for outcome assessments. If the experimental group used different doses, only the highest dose was extracted. If outcomes were presented at different time points, only the last time point was extracted. If the data were expressed graphically, we extracted it by use of digital ruler software. The mean value and standard deviation were extracted from the data of experimental and control groups of each comparison.

### Risk of Bias Assessment

The Collaborative Approach to Meta-Analysis and Review of Animal Data in Experimental Studies (CAMARADES) 10-item quality checklist was proposed by Macleod et al., (2004) for studying the efficacy of candidate drugs in experimental stroke. Two investigators independently assessed the methodological quality of the included studies based on the checklist as follows: (1) publication in a peer-reviewed journal; (2) statement of control of temperature; (3) randomization to treatment or control; (4) blinded induction of ischemia; (5) blinding of outcome assessment; (6) no obvious intrinsic neuroprotective effect of anesthetic; (7) appropriate animal model such as aged, diabetic, or hypertensive; (8) sample size estimation; (9) compliance with animal welfare regulations; (10) declared any potential conflict of interest. Each item of the 10-item scale contributed one point, and each study was given an aggregate quality score. Any disagreements were settled through consultation with the corresponding author.

### Statistical Analysis

The mean difference (MD) with 95% confidence intervals (CI) was used as a summary statistic when outcome measures in all studies applied the same scale. On the contrary, standardized mean difference (SMD) with 95% CI was used when the same outcome index was measured in various ways. Statistical heterogeneity was assessed by the I-square test. A random-effects model test was conducted when there was significant heterogeneity (I^2^ > 50%). Otherwise, the fixed-effects model test was adopted. Sensitivity analysis and subgroup analysis were carried out to find the origin of heterogeneity. A probability value of less than 0.05 was considered statistically significant. Data analyses were performed by Rev Man 5.3.

## Results

### Study Selection

**Figure 2:**
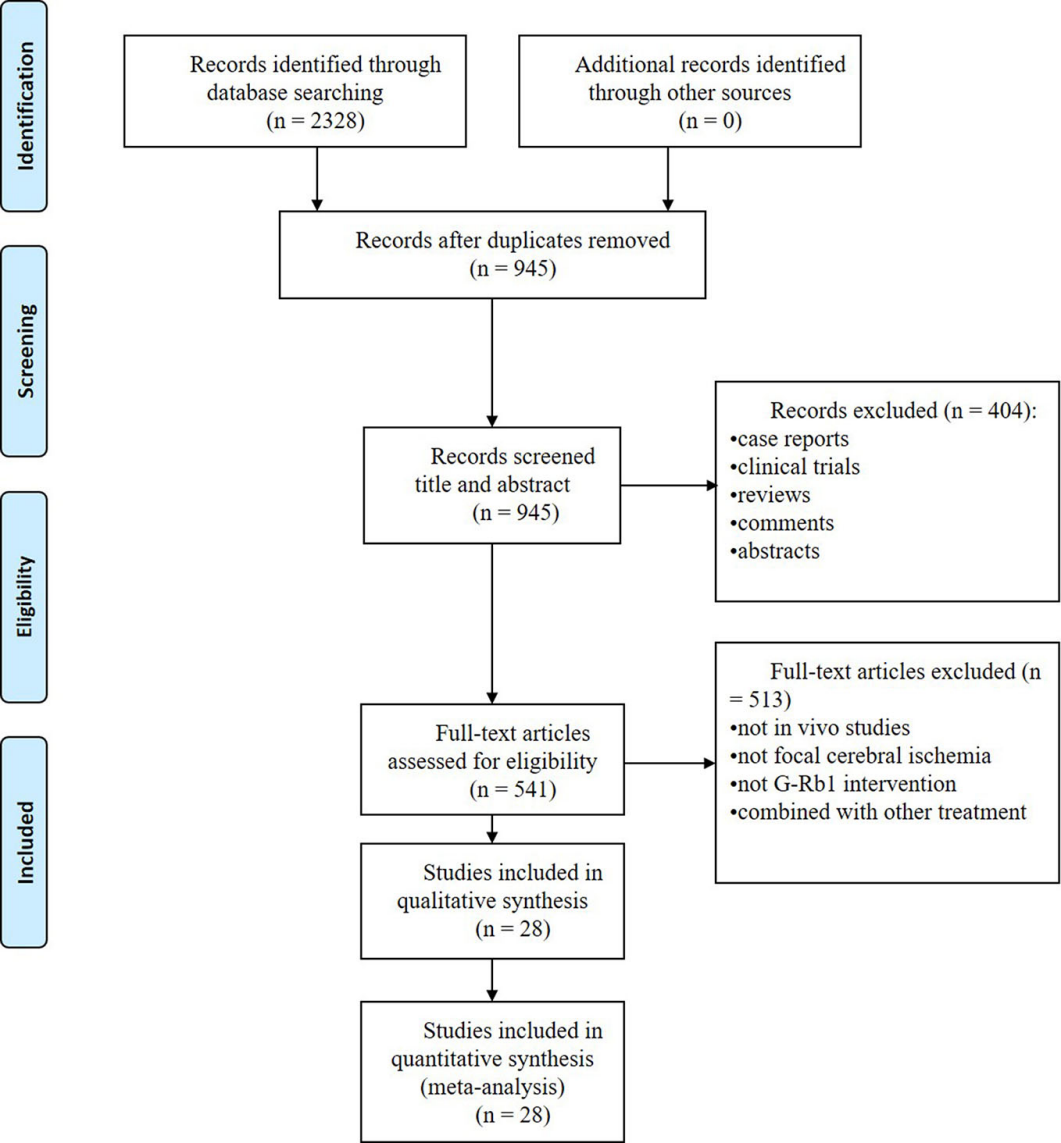
PRISMA flow chart of the search process.

We identified 2328 potentially relevant articles, and 1383 studies were removed because they were duplicates. After going through the titles and abstracts, 404 studies were excluded because they were case reports, clinical trials, reviews, comments or abstracts. Through reading the full text of the remaining 541 articles, 513 studies were further removed with at least one of reasons as follows: (1) not *in vivo* studies; (2) not focal cerebral ischemia; (3) not G-Rb1 intervention or combined with other treatment. Eventually, we included 28 studies (Zhang et al., 1998; Tao, 2004; Li et al., 2005; Ren, 2005; Fan, 2006; Zhang et al., 2006; Yoshikawa et al., 2008; Gao et al., 2010; Lu, 2010; Lu et al., 2011; Lv, 2011; Zhu et al., 2012; Jiang et al., 2013; Liu et al., 2013; Yu et al., 2013; Liu et al., 2014; Zeng et al., 2014; Chen et al., 2015; Dai et al., 2016; He et al., 2016; Lv et al., 2016; Dong et al., 2017; Gao, 2017; Li et al., 2018; Liu et al., 2018; Xu et al., 2018; Sun and Xiu, 2018; Shi et al., 2019) in the final analysis. The process of screening is summarized in a flow diagram (**Figure 2**).

### Study Characteristics

Twenty-eight studies with 937 animals were identified for analysis, among which 559 were included in experimental groups while 378 in control groups. Twelve studies were published in English and the remainder in Chinese. The animal species included Sprague-Dawley (SD) rats (Tao, 2004; Li et al., 2005; Ren, 2005; Fan, 2006; Lu, 2010; Lu et al., 2011; Zhu et al., 2012; Jiang et al., 2013; Liu et al., 2013; Yu et al., 2013; Liu et al., 2014; Dai et al., 2016; He et al., 2016; Li et al., 2018; Sun and Xiu, 2018; Xu et al., 2018; Shi et al., 2019), Wistar rats (Gao et al., 2010; Liu et al., 2018), stroke-prone spontaneously hypertensive rats (SHR-SP) (Zhang et al., 1998; Zhang et al., 2006), C57BL/6 mice (Zeng et al., 2014; Lv et al., 2016; Dong et al., 2017; Gao, 2017), BALB/c mice (Lv, 2011), Institute of Cancer Research (ICR) mice (Chen et al., 2015) and Cynomolgus monkeys (Yoshikawa et al., 2008). Chloral hydrate was used in 18 studies (Tao, 2004; Li et al., 2005; Ren, 2005; Fan, 2006; Lu, 2010; Lu et al., 2011; Lv, 2011; Jiang et al., 2013; Liu et al., 2013; Yu et al., 2013; Liu et al., 2014; Dai et al., 2016; He et al., 2016; Lv et al., 2016; Li et al., 2018; Liu et al., 2018; Xu et al., 2018; Sun and Xiu, 2018) to induce anesthesia, pentobarbital sodium in 5 studies (Gao et al., 2010; Zhu et al., 2012; Zeng et al., 2014; Chen et al., 2015; Shi et al., 2019), halothane in 2 studies (Zhang et al., 1998; Zhang et al., 2006), tribromoethanol in 1 study, ketamine and medetomidine in 1 study (Yoshikawa et al., 2008) and the remaining study did not report (Dong et al., 2017).

According to the pre-established inclusion and exclusion criteria, studies included in this systematic review generally show that: the baseline status of experimental animals remained consistent in each independent study; the animal subjects had the same disease model of focal cerebral ischemia that was induced by middle cerebral artery occlusion; all animal subjects in experimental groups received G-Rb1 monotherapy while those in control groups received normal saline or no treatment.

The assessment of NFS were different, as 15 studies adopted Zea longa (ZL) criterion (Tao, 2004; Ren, 2005; Fan, 2006; Lv, 2011; Liu et al., 2013; Yu et al., 2013; Liu et al., 2014; Zeng et al., 2014; Chen et al., 2015; Dai et al., 2016; He et al., 2016; Li et al., 2018; Liu et al., 2018; Xu et al., 2018; Sun and Xiu, 2018), 4 studies adopted the modified neurological severity score (mNSS) (Gao et al., 2010; Lu, 2010; Zhu et al., 2012; Shi et al., 2019), 2 studies adopted a water maze test (Zhang et al., 1998; Zhang et al., 2006), 2 studies adopted Bederson score (Jiang et al., 2013; Dong et al., 2017), and the remaining 3 studies adopted a rotarod test and beam walking performance (Gao, 2017), an 18-point scoring system (Lv et al., 2016) (Xing et al., 2008) and a standardized score (Yoshikawa et al., 2008) (Kito et al., 2001). For IV, 19 studies adopted triphenyl tetrazolium chloride (TTC) staining (Tao, 2004; Li et al., 2005; Ren, 2005; Zhang et al., 2006; Lu et al., 2011; Lv, 2011; Zhu et al., 2012; Jiang et al., 2013; Liu et al., 2013; Liu et al., 2014; Zeng et al., 2014; Chen et al., 2015; Dai et al., 2016; He et al., 2016; Lv et al., 2016; Dong et al., 2017; Li et al., 2018; Liu et al., 2018; Xu et al., 2018), 1 study used an MRI scan (Fan, 2006), 2 studies adopted hematoxylin-eosin (HE) staining (Yoshikawa et al., 2008; Lu, 2010), while 1 study did not report (Zhang et al., 1998). The included studies also reported neurogenesis related indicators [Brain derived neurotrophic factor (BDNF) (Gao et al., 2010; Jiang et al., 2013), Gap-43 (Jiang et al., 2013; Gao, 2017), Nestin-positive cells (Gao et al., 2010)], apoptosis related indicators [Nissl-positive cells (Zhang et al., 1998; Fan, 2006; Zhang et al., 2006; Lu, 2010; Lu et al., 2011), TUNEL-positive cells (Ren, 2005; Zhang et al., 2006; Yoshikawa et al., 2008; Zhu et al., 2012; Liu et al., 2018), Caspase-3 (Fan, 2006; Gao et al., 2010; Zeng et al., 2014; Lv et al., 2016), Bcl-x_l_ (Zhang et al., 2006)], inflammation related indicators [IL-1 (Jiang et al., 2013; Liu et al., 2013), IL-6 (Zhu et al., 2012; Yu et al., 2013; Liu et al., 2018), TNF-α (Zhu et al., 2012; Jiang et al., 2013; Yu et al., 2013; Liu et al., 2018), high-mobility group box 1 (HMGB1) (Liu et al., 2018), NF-κB (Zhu et al., 2012; Zeng et al., 2014; Lv et al., 2016; Liu et al., 2018)], oxidative stress related indicators [malondialdehyde (MDA) (Zeng et al., 2014; Dai et al., 2016; Dong et al., 2017), superoxide dismutase (SOD) (Zeng et al., 2014; Dai et al., 2016; Xu et al., 2018), glutathione (GSH) (Dong et al., 2017), reactive oxygen species (ROS) (Lv, 2011), nitric oxide (NO) (Lv et al., 2016; Dong et al., 2017; Liu et al., 2018), nicotinamide adenine dinucleotide phosphate oxidase (NOX) (Chen et al., 2015; Dong et al., 2017; Shi et al., 2019)], BWC (Ren, 2005; Yoshikawa et al., 2008; Chen et al., 2015; Dong et al., 2017; Shi et al., 2019), Evans blue content (Chen et al., 2015; Li et al., 2018), glucose transporters (GLUT) (Tao, 2004; Li et al., 2005) and CBF (Dai et al., 2016; He et al., 2016; Lv et al., 2016).

The details of the analyzed studies were presented in **Table 1**. The statement of the characteristics of G-Rb1 was presented in **Table 2**.

**Table 1:** Basic characteristics of the included studies.

Study	Gender, species, number	Weight	Anesthetic	Model (method)	Method of treatment			Outcome measure	Intergroup differences
					G-Rb1	Control	Administration		
Chen et al., 2015	Male, ICR mice (8/8/8/8)	25–30 g	Pentobarbital sodium	MCAO(t) 1h (ZL)	40 mg/kg	NS (same volume)	3h after reperfusion; i.p.; 1 time	1.NFS (ZL, 48h) 2.IV (TTC, 48h) 3.Evans blue content (48h) 4.BWC (48h) 5.NOX-1 mRNA(24h) 6.NOX-4 mRNA(24h) 7.NOX activity (24h)	1.P < 0.01 2.P < 0.01 3.P < 0.01 4.P < 0.01 5.P < 0.05 6.P < 0.01 7.P < 0.05
Dai et al., 2016	Male, SD rats (12/12)	220 ± 10 g	10% Chloralhydrate (400 mg/kg)	MCAO(t) 2h (ZL)	40 mg/kg	NS (same volume)	immediately after occlusion; i.p.; 1 time	1.NFS (ZL, 24h) 2.IV (TTC, 24h) 3.MDA (24h) 4.SOD (24h) 5.cerebral blood flow (24h)	1.P < 0.05 2.P < 0.05 3.P < 0.05 4.P < 0.05 5.P < 0.05
Dong et al., 2017	NR, C57BL/6J mice (8/8/8/8) Aged model (24 m)	NR	NR	MCAO(t) 1h (ZL)	10 mg/kg	NS (same volume)	1yr before occlusion; p.o.; every 3d for 1yr	1.NFS (Bederson, 7d) 2.IV (TTC, 7d) 3.BWC (7d) 4.GSH (7d) 5.MDA (7d) 6.NO (7d) 7.NOX-1 mRNA(24h) 8.NOX-4 mRNA(24h) 9.NOX activity(24h)	1.P < 0.01 2.P < 0.01 3.P < 0.05 4.P < 0.01 5.P < 0.01 6.P < 0.01 7.P < 0.01 8.P < 0.01 9.P < 0.01
Fan, 2006	Male, SD rats (16/16)	230–270 g	10% Chloralhydrate (350 mg/kg)	MCAO(t) 2h (ZL)	20 mg/kg	NS (same volume)	1h after occlusion; i.p.; once daily for 5 d	1. NFS (ZL, 24h) 2. IV (MRI,24h) 3.Caspase-3 positive cells (24h) 4.Nissl-positive cells (24h)	1.P < 0.05 2.P < 0.05 3.P < 0.01 4.P < 0.01
Gao, 2017	Male, C57BL/6 mice (8/8)	25-30g	Tribromoethanol (0.4 g/kg)	MCAO(p) Coagulated	50 mg/kg	NS (same volume)	24h after occlusion; i.p.; once daily for 14 d	1.NFS (Rotarod test, 28d) 2.NFS (Beam walking performance, 28d) 3.GAP-43 (28d)	1.P < 0.05 2.P < 0.05 3.P < 0.05
Gao et al., 2010	Male, Wistar rats (28/28)	250–300 g	1% Pentobarbital sodium (30 mg/kg)	MCAO(t) 2h (ZL)	40 mg/kg	NS (same volume)	immediately after reperfusion; i.p.; 1 time	1.NFS (mNSS, 5d) 2.Nestin-positive cells (5d) 3.Caspase-3 (5d) 4.BDNF (5d)	1.P < 0.05 2.P < 0.05 3.P < 0.05 4.P < 0.05
He et al., 2016	Male, SD rats (6/6)	220 ± 10 g	10% Chloralhydrate	MCAO(t) 2h (ZL)	40 mg/kg	NS (same volume)	immediately after reperfusion; i.p.; 1 time	1.NFS (ZL, 24h) 2.IV (TTC, 24h) 3.cerebral blood flow (24h)	1.P < 0.05 2.P < 0.05 3.P < 0.05
Jiang et al., 2013	Male, SD rats (10/10)	280–350 g	10% Chloralhydrate (0.35 g/kg)	MCAO(t) (3h) (ZL)	93.75 mg/kg	No Treatment	3d before occlusion; i.g.; 3 times daily for 3 d	1.NFS (Bederson,24h) 2.IV (TTC, 24h) 3.IL-1 (24h) 4.TNF-α (24h) 5.BDNF (24h) 6.GAP-43 (24h)	1. NR 2.P < 0.05 3.P < 0.05 4.P < 0.05 5.P < 0.05 6.P < 0.05
Jiang et al., 2013	Male, SD rats (10/10)	280–350 g	10% Chloralhydrate (0.35 g/kg)	MCAO(t) (3h) (ZL)	93.75 mg/kg	No Treatment	3h after occlusion; i.g.; 3 times daily for 3 times	1.IV (TTC, 24h) 2.IL-1 (24h) 3.TNF-α (24h) 4.BDNF (24h) 5.GAP-43 (24h)	1.P < 0.05 2.P < 0.05 3.P < 0.05 4.P < 0.05 5.P < 0.05
Li et al., 2005	Male, SD rats (15/15)	300 ± 10 g	10% Chloralhydrate (0.3 ml/100 g)	MCAO(t) 1h (ZL)	20 mg/kg	NS (same volume)	5d before occlusion; i.p.; once daily for 5d	1.IV (TTC, 5h) 2.GLUT3 protein/mRNA (5h)	1.P < 0.01 2.P < 0.05
Li et al., 2018	Male, SD rat (20/20)	250–280 g	10% Chloralhydrate (3 ml/kg).	MCAO(t) 2h (ZL)	25 g/kg,	NS (same volume)	3d before occlusion; i.p.; once daily for 10d	1.NFS (ZL,7d) 2. Evans blue content (7d) 3. IV (TTC,7d) 4. AQP4 protein/mRNA (7d) 5. Cx43 protein/mRNA (7d)	1.P < 0.01 2.P < 0.05 3.P < 0.01 4.P < 0.01 5.P < 0.01
Liu et al., 2018	Male, Wistar rats (8/8/8/8)	270-330 g	10% Chloralhydrate (350 mg/kg)	MCAO(t) 2h (ZL)	200 mg/kg	NS (same volume)	immediately after occlusion; i.v.	1.NFS (ZL, 24h) 2.IV (TTC, 24h) 3.TUNEL-positive cells (24h) 4.TNF-α (24h) 5.IL-6 (24h) 6.HMGB1 (24h) 7.NO (24h) 8.iNOS (24h) 9.NF-κB p65 (24h)	1.P < 0.01 2.P < 0.01 3.P < 0.01 4.P < 0.01 5.P < 0.01 6.P < 0.01 7.P < 0.01 8.P < 0.01 9.P < 0.01
Liu et al., 2013	Male, SD rats (12/12/12/12)	260–300 g	10% Chloralhydrate (350 mg/kg)	MCAO(t) 2h (ZL)	80 mg/kg	NS (same volume)	immediately after occlusion; i.p.; 1 time	1.NFS (ZL, 24h) 2.IV (TTC, 24h) 3.IL-1β (24h)	1.P < 0.01 2.P < 0.01 3.P < 0.01
Liu et al., 2014	Male, SD rats (8/8)	260–300 g	10% Chloralhydrate (350 mg/kg)	MCAO(t) 2h (ZL)	40 mg/kg	NS (4ml/kg);	immediately after occlusion; i.p.; 1 time	1.NFS (ZL, 24h) 2.IV (TTC, 24h)	1.P < 0.05 2.P < 0.05
Lu, 2010	Male, SD rats (12/12)	240–260g	10% Chloralhydrate (350 mg/kg)	MCAO(t) 1.5h (ZL)	12.5 mg/kg	NS (same volume)	immediately after occlusion; i.n.; once daily for 14 d	1.NFS (mNSS, 14d) 3.IV (HE, 14d) 3.Nissl-positive cells	1.P > 0.05 2.P < 0.05 3.NR
Lu et al., 2011	Male, SD rats (5/4)	250 ± 10 g	Chloralhydrate (400 mg/kg)	MCAO(t) 1.5h (ZL)	12.5 mg/kg	NS (same volume)	immediately after occlusion; i.n.; 1 time	1.IV (TTC, 24h) 2.LC3II (24h) 3.Baclin-1 (24h) 4.Nissl-positive cells	1.P < 0.01 2.P < 0.05 3.P < 0.05 4.NR
Lv, 2011	Male, BALB/C mice (30/30)	20 ± 2 g	3% Chloralhydrate (10 ml/kg)	MCAO(t) 1h (ZL)	10 mg/kg	NS (same volume);	30min after occlusion; i.p.; 1 time	1.NFS (ZL, 24h) 2.IV (TTC, 24h) 3.ROS (24h)	1.P < 0.05 2.P < 0.05 3.P < 0.05
Lv et al., 2016	Male, C57BL/6J mice (24/24)	18-22 g	4% Chloralhydrate (10 ml/kg)	MCAO(t) 1h (ZL)	3.33 mg/kg	No Treatment	1h after occlusion; i.v.; 1 time	1.NFS (24h) 2.IV (TTC, 24h) 3.NO (24h) 4.Caspase-3 (24h) 5.NF-κB p-p65/NF-κB p65 (24h) 6.cerebral blood flow (24h)	1.P < 0.01 2.P < 0.01 3.P < 0.05 4.P < 0.01 5.P < 0.05 6.P < 0.01
Ren, 2005	Male, SD rats (18/18/18)	220—280 g	3.5% Chloralhydrate (1 ml/100 g)	MCAO(t) 2h (ZL)	90 mg/kg	NS (same volume)	7d before occlusion; i.p.; once daily for 7d	1.NFS (ZL, 48h) 2.IV (TTC, 48h) 3.BWC (48h) 4.TUNEL-positive cells (48h)	1.P < 0.05 2.P < 0.01 3.P < 0.01 4.P < 0.01
Tao, 2004	Male, SD rats (15/12)	300 ± 10 g	10% Chloralhydrate (0.3 ml/100 g)	MCAO(t) 1h (ZL)	20 mg/kg	NS (same volume)	5d before occlusion; i.p.; once daily for 5d	1.NSF (ZL, 5h) 2.IV (TTC,5h) 3.GLUT1 protein/mRNA (5h) 4.GLUT3 protein/mRNA (5h)	1.P < 0.05 2.P < 0.05 3.P < 0.05 4.P < 0.05
Yoshikawa et al., 2008	Male, Cynomolgus monkeys (4/4)	4 – 6 kg	Ketamine (25 mg/kg) and Medetomidine (50 μg/kg)	MCAO(p) Thrombo-embolic	300 μg/kg	NS (same volume)	7d before occlusion; i.v.; once daily for 8d	1.NFS (Kito,7d) 2.IV (HE,7d)	1.P > 0.05 2.P > 0.05
Yoshikawa et al., 2008	Male, Cynomolgus monkeys (5/5)	4 – 6 kg	Ketamine (25 mg/kg) and Medetomidine (50 μg/kg)	MCAO(p) Thrombo-embolic	300 μg/kg	NS (same volume)	2d before occlusion; i.v.; once daily for 10d	1.NFS (Kito,7d) 2.IV (HE,7d)	1.P > 0.05 2.P > 0.05
Yoshikawa et al., 2008	Male, Cynomolgus monkeys (5/7)	4 – 6 kg	Ketamine (25 mg/kg) and Medetomidine (50 μg/kg)	MCAO(p) Thrombo-embolic	300 μg/kg	NS (same volume)	7d before occlusion; i.v.; once daily for 8d	1.BWC (7d) 2.NeuN-positive cells (7d) 3.TUNEL-positive cells (7d)	1.P > 0.05 2.P < 0.05 3.P < 0.01
Yu et al., 2013	Male, SD rats (12/12/12/12)	250-300 g	10% Chloralhydrate (350 mg/kg)	MCAO(t) 2h (ZL)	40 mg/kg	NS (same volume)	immediately after reperfusion; i.p.; 1 time	1.NFS (ZL,24h) 2.TNF-α (24h) 3.IL-6 (24h) 4.p-JAK 2 positive cell (24h) 5.p-STAT 3 positive cell (24h)	1.P < 0.05 2.P < 0.01 3.P < 0.01 4.P < 0.05 5.P < 0.01
Zeng et al., 2014	Male, C57BL/6 mice (6/6)	25–30 g	Pentobarbital sodium (50 mg/kg)	MCAO(t) 2h (ZL)	40 mg/kg	NS (same volume)	2h after occlusion; i.p.; twice daily for 2d	1.NFS (ZL,24h) 2.IV (TTC,24h) 3.MDA (24h) 4.Caspase-3 (24h) 5.Trx-1 (24h) 6.SOD-1 (24h) 7.HSP70 (24h) 8.Akt (24h) 9.p-NF-κB/NF-κB (24h)	1.P < 0.05 2.P < 0.01 3.P < 0.01 4.P < 0.01 5.P < 0.01 6.P < 0.01 7.P < 0.05 8. P < 0.01 9. P < 0.01
Zhang et al., 2006	NR, SHR-SP rats (8/8/8)	250–300 g	1.5% Halothane	MCAO(p) coagulated	200 μg/kg	NS (same volume)	immediately after occlusion; i.v.; once daily for 4 wk	1.NFS (Water Maze Test, 4w) 2.IV (TTC, 4w) 3.TUNEL-positive cells (4w) 4.Nissl-positive cells	1.P < 0.01 2.P < 0.01 3.P < 0.01 4.NR
Zhang et al., 2006	NR, SHR-SP rats (7/7/7)	250–300 g	1.5% Halothane	MCAO(p) coagulated	200 μg/kg	NS (same volume)	2h after occlusion; i.v.; 1 time	1.IV (TTC, 24h) 2.Bcl-xl (24h)	1.P < 0.05 2.P < 0.01
Zhang et al., 1998	NR, SHR-SP rats (8/8/8/8/8)	250–320 g	1.5% Halothane	MCAO(p) coagulated	20 μg/kg	NS (same volume)	2h before occlusion; i.c.v.; once daily for 4 wk	1.NFS (Water Maze Test, 4w) 2.IV (4w) 3.Nissl-positive cells	1.P < 0.05 2.P > 0.05 3.NR
Zhang et al., 1998	NR, SHR-SP rats (8/8/8/8)	250–320 g	1.5% Halothane	MCAO(p) coagulated	20 μg/kg	NS (same volume)	immediately after occlusion; i.c.v.; once daily for 4 wk	1.NFS (Water Maze Test, 4w) 2.IV (4w)	1.P < 0.01 2.P > 0.05
Zhu et al., 2012	Male, SD rat (12/12)	220–250 g	Pentobarbital sodium (40 mg/kg)	MCAO(t) 2h (ZL)	12.5 mg/kg	NS (same volume)	7d before occlusion; i.n.; once daily for 7d	1.NFS (mNSS, 72h) 2.IV (TTC, 24h) 3.TUNEL-positive cells (24h) 4.TNF-α protein/mRNA (72h) 5.IL-6 protein/mRNA (72h) 6.NF-κB p65 (72h) 7.p-NF-κB p65 (72h)	1.P < 0.05 2.P < 0.01 3.P < 0.01 4.P > 0.05 5.P > 0.05 6.P < 0.01 7.P > 0.05
Sun and Xu, 2018	Male, SD rat (20/20)	180–200 g	10% Chloralhydrate (350 mg/kg)	MCAO(t) 2h (ZL)	20 mg/kg	NS (same volume)	immediately after occlusion; i.p. once daily for 7d	1.NFS (ZL,24h) 2.PI3K protein/mRNA (7d) 3.p-AKT protein/mRNA (7d)	1.P < 0.01 2.P < 0.01 3.P < 0.01
Shi et al., 2019	Male, SD rat (10/10)	350-450 g	3% Pentobarbital sodium (65 mg/kg)	MCAO(t) 0.5h (ZL)	20 mg/kg	No Treatment	1h after occlusion; i.p.; 1time	1.NFS (mNSS,8h) 2.BWC (8h) 3.Cx40 protein (8h) 4.NOX activity (8h)	1.P < 0.05 2.P < 0.05 3.P < 0.05 4.P < 0.05
Xu et al., 2019	Male, SD rat (12/12)	280 ± 20 g	10% Chloralhydrate (350 mg/kg)	MCAO(t) 2h (ZL)	40 mg/kg	NS (same volume)	immediately after occlusion; i.p. 1 time	1.NFS (ZL,24h) 2.IV (TTC, 4h) 3.SOD (24h)	1.P < 0.05 2.P < 0.05 3.P < 0.05

SD, Sprague—Dawley; SHR-SP, Spontaneously Hypertensive Rats-Stroke Prone; NFS, neurological function score; IV, infarct volume; ZL, Zea Longa; MCAO, middle cerebral artery occlusion; h, hour; d, day; wk, week; yr, year; NR, not report; G-Rb1, Ginsenoside-Rb1; mNSS, modified neurological severity scores; TTC, 2, 3, 5-triphenyltetrazolium chloride; HE, hematoxylin-eosin; BWC, brain water content; NS, normal saline; i.p., intraperitoneal; i.n., intranasal; i.c.v., intralateroventricular; i.v., intravenous; p.o., per os; t, temporary; p, permanent.

**Table 2:** Statement of the characteristics of G-Rb1.

Study	Herb source	Dose of G-Rb1	Purity	Approach to achieving	Quality control (lot number)	Chemical analysis
Chen et al., 2015	Panax ginseng C.A. Meyer	intraperitoneal injected at 40 mg/kg	not reported	not reported	not reported	not included
Dai et al., 2016	Panax ginseng C.A. Meyer	intraperitoneal injected at 40 mg/kg	≥ 98%	Shanghai Tauto Biotech Co., Ltd., Shanghai, China	reported (10072432)	included
Dong et al., 2017	Panax ginseng C.A. Meyer	oral gavage at 10 mg/kg	92.6%	National Institute for the Control of Pharmaceutical and Biological Produces, Beijing, China	reported (110704-200921)	included
Fan, 2006	Panax ginseng C.A. Meyer	intraperitoneal injected at 20 mg/kg	> 99.5%	Department of Organic Chemistry, College of Basic Medical Sciences, Jilin University	not reported	not included
Gao, 2017	Panax ginseng C.A. Meyer	dissolved in saline to 5 mg/ml for preparation; intraperitoneal injected at 50 mg/kg	> 98.5%	Nanjing Ze-Long Pharmaceutical Co., Ltd., Nanjing, China	reported (141109)	included
Gao et al., 2010	Panax ginseng C.A. Meyer	dissolved in saline to 40 mg/ml for preparation; intraperitoneal injected at 40 mg/kg	> 98%	National Institute for the Control of Pharmaceutical and Biological Produces, Beijing, China	reported (110704-200318)	included
He et al., 2016	Panax ginseng C.A. Meyer	intraperitoneal injected at 40 mg/kg	not reported	not reported	not reported	not included
Jiang et al., 2013	Panax ginseng C.A. Meyer	dissolved in saline to 7.5 mg/ml for preparation; intragastric administrated at 93.75 mg/kg	≥ 98%	Nanjing Ze-Long Pharmaceutical Co., Ltd., Nanjing, China	reported (ZL20100705A)	included
Li et al., 2005	Panax ginseng C.A. Meyer	intraperitoneal injected at 20 mg/kg	> 99.5%	Department of Organic Chemistry, College of Basic Medical Sciences, Jilin University	not reported	not included
Li et al., 2018	Panax ginseng C.A. Meyer	dissolved in saline to 10 mg/ml for preparation; intraperitoneal injected at 25 g/kg	≥ 98%	Shanghai Tauto Biotech Co., Ltd., Shanghai, China	reported (10072432)	included
Liu et al., 2018	Panax ginseng C.A. Meyer	intravenously injected at 200 mg/kg	not reported	not reported	not reported	not included
Liu et al., 2013	Panax ginseng C.A. Meyer	intraperitoneal injected at 80 mg/kg	≥ 98%	Shanghai Tauto Biotech Co., Ltd., Shanghai, China	reported (10072432)	included
Liu et al., 2014	Panax ginseng C.A. Meyer	intraperitoneal injected at 40 mg/kg	≥ 98%	Shanghai Tauto Biotech Co., Ltd., Shanghai, China	reported (10072432)	included
Lu, 2010	Panax ginseng C.A. Meyer	dissolved in saline to 1 mg/ml for preparation; intranasal administrated at 12.5 mg/kg	≥ 98%	Nanjing Ze-Long Pharmaceutical Co., Ltd., Nanjing, China	reported (ZL20100705A)	included
Lu et al., 2011	Panax ginseng C.A. Meyer	dissolved in saline to 1 mg/ml for preparation; intranasal administrated at 12.5 mg/kg	≥ 98%	Nanjing Ze-Long Pharmaceutical Co., Ltd., Nanjing, China	reported (ZL20100705A)	included
Lv, 2011	Panax ginseng C.A. Meyer	dissolved in saline to 20 mmol/ml for preparation; intraperitoneal injected at 10 mg/kg	≥ 98%	Sigma, USA	reported (Y0001347)	included
Lv et al., 2016	Panax ginseng C.A. Meyer	intravenously injected at 3.33 mg/kg	> 98.5%	Nanjing Ze-Long Pharmaceutical Co., Ltd., Nanjing, China	reported (141109)	included
Ren, 2005	Panax ginseng C.A. Meyer	dissolved in saline to 10 mg/ml for preparation; intraperitoneal injected at 90 mg/kg	92%	Yunnan Phytopharmaceutical Co., Ltd., China	not reported	not included
Tao, 2004	Panax ginseng C.A. Meyer	intraperitoneal injected at 20 mg/kg	> 99.5%	Department of Organic Chemistry, College of Basic Medical Sciences, Jilin University	not reported	not included
Yoshikawa et al., 2008	Panax ginseng C.A. Meyer	dissolved in saline to 300 µg/ml for preparation; intravenously injected at 300 µg/kg	not reported	repeated-column chromatography	not reported	not included
Yu et al., 2013	Panax ginseng C.A. Meyer	intraperitoneal injected at 40 mg/kg	≥ 98%	Nanjing Ze-Long Pharmaceutical Co., Ltd., Nanjing, China	reported (ZL20100705A)	included
Zeng et al., 2014	Panax ginseng C.A. Meyer	intraperitoneal injected at 40 mg/kg	99.1%	Kunming Pharmaceutical Corporation, Kunming, China	not reported	not included
Zhang et al., 2006	Panax ginseng C.A. Meyer	intravenously injected at 200 µg/kg	> 98%	repeated-column chromatography	not reported	not included
Zhang et al., 1998	Panax ginseng C.A. Meyer	intralateroventricular injected at 20 μg/kg	> 98%	not reported	not reported	not included
Zhu et al., 2012	Panax ginseng C.A. Meyer	dissolved in saline to 160 mg/ml for preparation; intranasal administrated at 12.5 mg/kg	≥ 98%	Nanjing Ze-Long Pharmaceutical Co., Ltd., Nanjing, China	reported (ZL20100705A)	included
Sun and Xu, 2018	Panax ginseng C.A. Meyer	intraperitoneal injected at 20 mg/kg	≥ 98%	Chengdu Pufei De Biotech Co., Ltd., Chengdu, China	reported (JOT10297)	included
Shi et al., 2019	Panax ginseng C.A. Meyer	intraperitoneal injected at 20 mg/kg	> 99%	Shanghai Bangde Biotech Co., Ltd., Shanghai, China	reported (B10759)	included
Xu et al., 2019	Panax ginseng C.A. Meyer	intraperitoneal injected at 40 mg/kg	≥ 98%	Shanghai Tauto Biotech Co., Ltd., Shanghai, China	reported (10073425)	included

### Risk of Bias

According to CAMARADES 10-item checklist (Macleod et al., 2004), the quality of included studies was evaluated as follows:

- Twenty-two studies were published in a peer-reviewed journal while the remaining 6 (Tao, 2004; Ren, 2005; Fan, 2006; Lu, 2010; Lv, 2011; Gao, 2017) were Master’s thesis or Ph.D. thesis.
- Twenty-one studies (Zhang et al., 1998; Tao, 2004; Ren, 2005; Fan, 2006; Zhang et al., 2006; Gao et al., 2010; Lu, 2010; Lu et al., 2011; Zhu et al., 2012; Liu et al., 2013; Yu et al., 2013; Liu et al., 2014; Zeng et al., 2014; Dai et al., 2016; He et al., 2016; Lv et al., 2016; Gao, 2017; Li et al., 2018; Liu et al., 2018; Xu et al., 2018; Shi et al., 2019) reported control of temperature, including control of rats’ anal temperature.
- Randomization allocation to groups was described in 22 studies (Tao, 2004; Li et al., 2005; Ren, 2005; Fan, 2006; Gao et al., 2010; Lu, 2010; Lu et al., 2011; Zhu et al., 2012; Liu et al., 2013; Yu et al., 2013; Liu et al., 2014; Zeng et al., 2014; Chen et al., 2015; Dai et al., 2016; He et al., 2016; Lv et al., 2016; Gao, 2017; Li et al., 2018; Liu et al., 2018; Xu et al., 2018; Sun and Xiu, 2018; Shi et al., 2019).
- None of the studies reported blinded induction of ischemia.
- Blinded outcome assessment was reported in 11 studies (Zhang et al., 1998; Zhang et al., 2006; Yoshikawa et al., 2008; Gao et al., 2010; Lu, 2010; Zhu et al., 2012; Liu et al., 2013; Dai et al., 2016; Liu et al., 2018; Xu et al., 2018; Shi et al., 2019).
- All studies except 1 (Dong et al., 2017) used anesthetic without significant intrinsic neuroprotective activity.
- Three studies (Zhang et al., 1998; Zhang et al., 2006; Dong et al., 2017) used an aged or hypertensive animal model.
- One study described sample size calculation (Shi et al., 2019).
- Nineteen studies (Zhang et al., 1998; Zhang et al., 2006; Yoshikawa et al., 2008; Gao et al., 2010; Lu et al., 2011; Zhu et al., 2012; Jiang et al., 2013; Zeng et al., 2014; Chen et al., 2015; Dai et al., 2016; He et al., 2016; Lv et al., 2016; Dong et al., 2017; Gao, 2017; Li et al., 2018; Liu et al., 2018; Sun and Xiu, 2018; Xu et al., 2018; Shi et al., 2019) gave an animal welfare statement.
- All included studies except 1 (Yoshikawa et al., 2008) mentioned a potential conflict of interests. The quality score of studies ranged from 2 to 7 (mean ± SD: 5.32 ± 1.32).

Summary of the methodological quality was presented in **Table 3**.

**Table 3:** Risk of bias of included studies according to CAMARADES checklist.

Study	(1)	(2)	(3)	(4)	(5)	(6)	(7)	(8)	(9)	(10)	Total
Chen et al., 2015	✓		✓			✓			✓	✓	5
Dai et al., 2016	✓	✓	✓		✓	✓			✓	✓	7
Dong et al., 2017	✓						✓		✓	✓	4
Fan, 2006		✓	✓			✓				✓	4
Gao, 2017		✓	✓			✓			✓	✓	5
Gao et al., 2010	✓	✓	✓		✓	✓			✓	✓	7
He et al., 2016	✓	✓	✓			✓			✓	✓	6
Jiang et al., 2013	✓					✓			✓	✓	4
Li et al., 2005	✓		✓			✓				✓	4
Li et al., 2018	✓	✓	✓			✓			✓	✓	6
Liu et al., 2018	✓	✓	✓		✓	✓			✓	✓	7
Liu et al., 2013	✓	✓	✓		✓	✓				✓	6
Liu et al., 2014	✓	✓	✓			✓				✓	5
Lu, 2010		✓	✓		✓	✓				✓	5
Lu et al., 2011	✓	✓	✓			✓			✓	✓	6
Lv, 2011						✓				✓	2
Lv et al., 2016	✓	✓	✓			✓			✓	✓	6
Ren, 2005		✓	✓			✓				✓	4
Tao, 2004		✓	✓			✓				✓	4
Yoshikawa et al., 2008	✓				✓	✓			✓		4
Yu et al., 2013	✓	✓	✓			✓				✓	5
Zeng et al., 2014	✓	✓	✓			✓			✓	✓	6
Zhang et al., 2006	✓	✓			✓	✓	✓		✓	✓	7
Zhang et al., 1998	✓	✓			✓	✓	✓		✓	✓	7
Zhu et al., 2012	✓	✓	✓		✓	✓			✓	✓	7
Sun and Xu, 2018	✓		✓			✓			✓	✓	5
Shi et al., 2019	✓	✓	✓		✓	✓		✓	✓	✓	8
Xu et al., 2019	✓	✓	✓		✓	✓			✓	✓	7

Studies fulfilling the criteria of: (1) publication in a peer-reviewed journal; (2) statement of control of temperature; (3) randomization of treatment or control; (4) blinded induction of ischemia; (5) blinding of outcome assessment; (6) no obvious intrinsic neuroprotective effect of anesthetic; (7) appropriate animal model (aged, diabetic, or hypertensive); (8) sample size estimation; (9) compliance with animal welfare regulations; (10) declared any potential conflict of interest.

### Effectiveness

#### NFS

**Figure 3:**
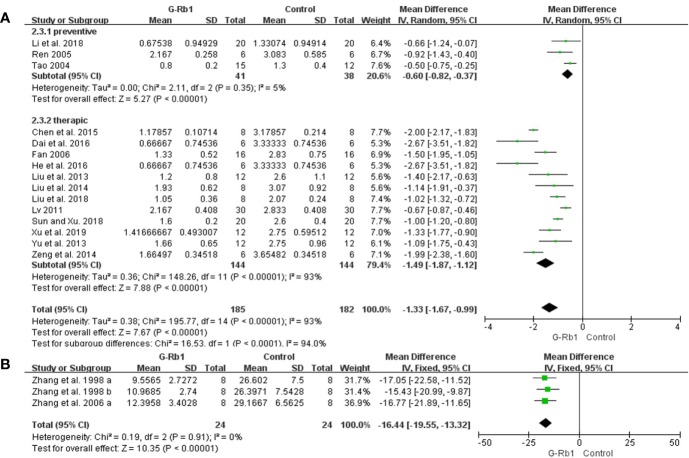
The pooled estimate of G-Rb1 on improving neurological function score according to: **(A)** ZL score; **(B)** Water maze test.

ZL score: Meta-analysis of 15 studies (Tao, 2004; Ren, 2005; Fan, 2006; Lv, 2011; Liu et al., 2013; Yu et al., 2013; Liu et al., 2014; Zeng et al., 2014; Chen et al., 2015; Dai et al., 2016; He et al., 2016; Li et al., 2018; Liu et al., 2018; Sun and Xiu, 2018; Xu et al., 2018) showed that G-Rb1 was significant for improving ZL scores compared with the control (n = 367, MD -1.33, 95% CI: -1.67 to -0.9, P < 0.01). Due to the obvious heterogeneity between studies (Chi-square = 195.77, df = 14, P < 0.01, I² = 93%), we conducted subgroup analysis based on the time of initial treatment, which showed a more meaningful result (Preventive: n = 79, MD -0.60, 95% CI: -0.82 to -0.37, P < 0.01; Heterogeneity: Chi-square = 2.11, df = 2, P > 0.1, I² = 5%; Therapeutic: n = 288, MD -1.49, 95% CI: -1.87 to -1.12, P < 0.01; Heterogeneity: Chi-square = 148.26, df = 11, P < 0.1, I² = 93%) (**Figure 3A**).

mNSS score: Meta-analysis of 4 studies (Gao et al., 2010; Lu, 2010; Zhu et al., 2012; Shi et al., 2019) showed a significant difference for improving mNSS scores (n = 70, MD –2.66, 95% CI: -5.10 to -0.22, P = 0.03; Heterogeneity: Chi-square = 159.68, df = 3, P < 0.1, I² = 98%). Heterogeneity existed in mNSS pooled data, probably coming from the different measurement and occlusion times.

Water maze test: Meta-analysis of 2 studies (3 comparisons) (Zhang et al., 1998; Zhang et al., 2006) indicated a significant benefit of G-Rb1 for improving water maze test compared with control group (n = 48, MD -16.44, 95% CI: -19.55 to -13.32, P < 0.01; Heterogeneity: Chi-square = 0.19, df = 2, P > 0.1, I² = 0%) (**Figure 3B**).

Bederson score: Dong et al. (2017) found that Bederson scores were lower in the G-Rb1 group than in the control group (n = 16, P < 0.01). Jiang et al. (2013) showed a similar result.

Others: Yoshikawa et al. (2008) showed that G-Rb1 reduced neurologic deficit score (n = 18, P < 0.05). Gao (Gao, 2017) reported that G-Rb1 increased the rotarod test score at 14 d and 28 d and improved the beam walking performance at 7 d, 14 d and 28 d after occlusion compared with the control (n = 16, P < 0.05). Lv et al., (2016) found that G-Rb1 could improve neurological deficit using an 18-point scoring system (n = 12, P < 0.01).

#### IV

**Figure 4:**
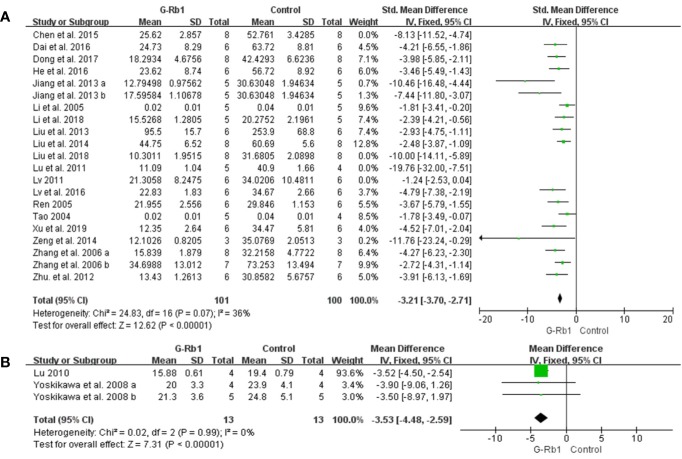
The pooled estimate of G-Rb1 on improving infarct volume according to: **(A)** TTC staining; **(B)** HE staining.

TTC staining: Meta-analysis of 19 studies with 21 comparisons (Tao, 2004; Li et al., 2005; Ren, 2005; Zhang et al., 2006; Lu et al., 2011; Lv, 2011; Zhu et al., 2012; Jiang et al., 2013; Liu et al., 2013; Liu et al., 2014; Zeng et al., 2014; Chen et al., 2015; Dai et al., 2016; He et al., 2016; Lv et al., 2016; Dong et al., 2017; Li et al., 2018; Liu et al., 2018; Xu et al., 2018) showed significant effects of G-Rb1 for reducing IV compared with control groups (n = 254, SMD = -3.15, 95% CI -3.61 to -2.70, P < 0.01; Heterogeneity: Chi-square = 59.36, df = 20, P < 0.1, I² = 66%). After the removal of 4 studies (Lu et al., 2011; Lv, 2011; Chen et al., 2015; Liu et al., 2018), the IV between the two groups had a significant difference and the heterogeneity was reduced (n = 201, SMD = -3.21, 95% CI -3.70 to -2.71, P < 0.01; Heterogeneity: Chi-square = 24.83, df = 16, P < 0.1, I² = 36%) (**Figure 4A**).

In order to identify the effective dose of G-Rb1 on experimental ischemic stroke, we conducted subgroup analysis. The results illustrated that there was a significant difference between 2 doses of G-Rb1 (subgroup heterogeneity: I^2^^=^ 72.6%). Intraperitoneal injection of G-Rb1 at ≥40mg/kg/d showed a relatively better effect for reducing IV (n = 86, SMD = -3.13, 95% CI -3.85 to -2.41, P < 0.01; Heterogeneity: Chi-square = 3.90, df = 6, P > 0.1, I² = 0%) compared with that at less than 40mg/kg/d (n = 19, SMD = -1.79, 95% CI -2.96 to -0.62, P < 0.01; Heterogeneity: Chi-square = 0, df = 1, P > 0.1, I² = 0%). The results illustrated that intraperitoneal injections of G-Rb1 at 40mg/kg/d could reduce IV effectively (**Supplementary Image 1**).

In terms of the treatment time after MCAO surgery, subgroup analysis showed a significant difference between the included studies (subgroup heterogeneity: I^2^ = 70.7%). As the treatment time increased, G-Rb1 was notably better at reducing IV at the time point of 24h to 7d (n = 140, SMD = -3.52, 95% CI -4.16 to -2.88, P < 0.01; Heterogeneity: Chi-square = 15.72, df = 11, P > 0.1, I² = 30%) compared with that at less than 24h (n = 19, SMD = -1.79, 95% CI -2.96 to -0.62, P < 0.01; Heterogeneity: Chi-square = 0, df = 1, P > 0.1, I² = 0%). However, the pooled data showed a similar effect between the data of 24h-7d and ≥7d (n = 42, SMD = -3.50, 95% CI -4.59 to -2.42) illustrating that G-Rb1 plays a neuroprotective effect mainly in the acute phase (24h-7d) of experimental ischemic stroke (**Supplementary Image 2**).

HE staining: Two studies with 3 comparisons (Yoshikawa et al., 2008; Lu, 2010) reported HE staining as an outcome measurement. Meta-analysis showed an obvious effect of G-Rb1 (n = 26, MD = -3.53, 95% CI -4.48 to -2.59, P < 0.01; Heterogeneity: Chi-square = 0.02, df = 2, P > 0.1, I² = 0%) (**Figure 4B**).

Others: One study (Fan, 2006) showed a beneficial effect of G-Rb1 for reducing IV according to MRI scans (n = 8, P < 0.05). One study with 2 comparisons (Zhang et al., 1998) did not mention the measuring method and showed no significant difference of G-Rb1 compared with the control (P > 0.05).

#### BWC and Blood-Brain Barrier Permeability

**Figure 5:**
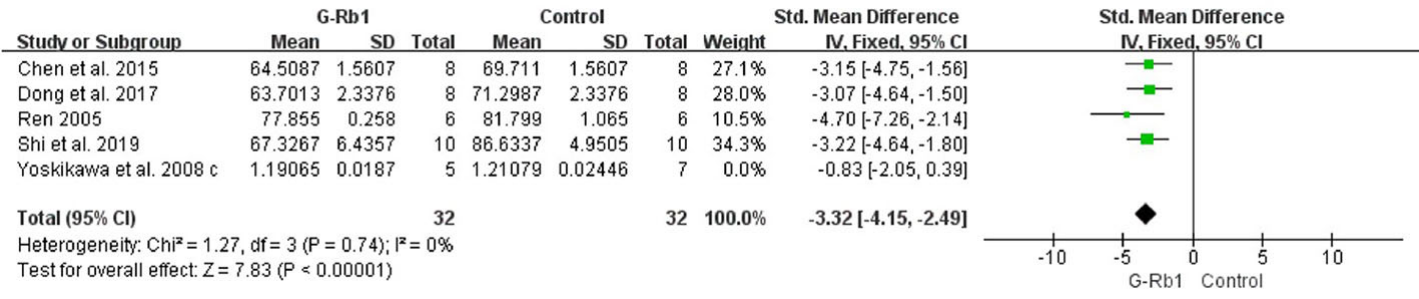
The pooled estimate of G-Rb1 on improving brain water content.

Five studies (Ren, 2005; Yoshikawa et al., 2008; Chen et al., 2015; Dong et al., 2017; Shi et al., 2019) investigated the effect of G-Rb1 on inhibiting brain edema following MCAO by testing BWC. Meta-analysis showed a significant reduction (n = 76, SMD = -2.53, 95% CI -3.21 to -1.84, P < 0.01; Heterogeneity: Chi-square = 12.19, df = 4, P < 0.1, I² = 67%). After removing 1 study (Yoshikawa et al., 2008), which used Cynomolgus monkeys as the animal model, the remaining 4 studies showed a significant difference when comparing G-Rb1 with the control (n = 64, SMD = -3.32, 95% CI -4.15 to -2.49, P < 0.01; Heterogeneity: Chi-square = 1.27, df = 3, P > 0.1, I² = 0%) (**Figure 5**).

Chen et al. (2015) (n = 16, P < 0.01) and Li et al. (2018) (n = 10, P < 0.05) reported reduced evans blue content after MCAO in a G-Rb1 group compared with the control.

#### Neuroprotective Mechanisms of G-Rb1

##### Neurogenesis

**Figure 6:**
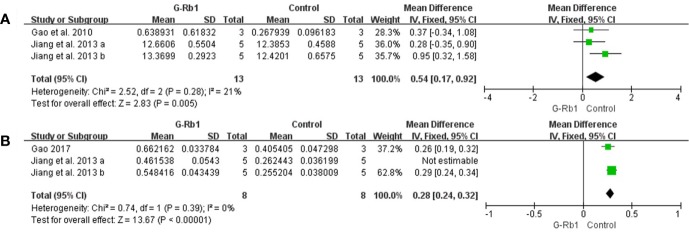
The pooled estimate of G-Rb1 on neurogenesis promotion according to: **(A)** BDNF level; **(B)** Gap-43 level.

Two studies with 3 comparisons (Gao et al., 2010; Jiang et al., 2013) assessed the BDNF level. Meta-analysis showed a significant increase in the G-Rb1 group (n = 26, MD = 0.54, 95% CI 0.17 to 0.92, P < 0.01; Heterogeneity: Chi-square = 2.52, df = 2, P > 0.1, I² =21%) (**Figure 6A**).

The pooled data of 3 comparisons (Jiang et al., 2013; Gao, 2017) showed a significant increase of Gap-43 by treatment with G-Rb1 (n = 26, MD = 0.25, 95% CI 0.19 to 0.31, P < 0.01; Heterogeneity: Chi-square = 5.85, df = 2, P < 0.1, I² = 66%). Removal of the outlier comparison Jiang et al. (2013) led to more homogeneous result (n = 16, MD = 0.28, 95% CI 0.24 to 0.32, P < 0.01; Heterogeneity: Chi-square = 0.74, df = 1, P > 0.1, I² = 0%) (**Figure 6B**).

Meanwhile Gao et al. (2010) (n = 10, P < 0.05), evaluating Nestin-positive cells, reported an obvious effect of G-Rb1 on increasing the number of neural precursors cells.

##### Anti-apoptosis

**Figure 7:**
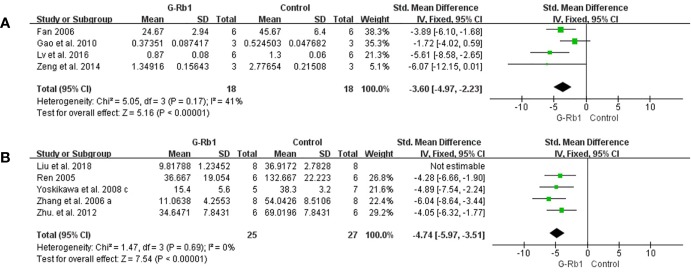
The pooled estimate of G-Rb1 on anti-apoptosis according to: **(A)** Caspase-3 level; **(B)** TUNEL-positive cells.

Four studies (Fan, 2006; Gao et al., 2010; Zeng et al., 2014; Lv et al., 2016) evaluated the anti- apoptosis effect of G-Rb1 by Caspase-3 levels compared with the control. Meta-analysis showed a significant reduction in the G-Rb1 group (n = 36, SMD = -3.60, 95% CI -4.97 to -2.23, P < 0.01; Heterogeneity: Chi² = 5.05, df = 3, P > 0.1, I² = 41%) (**Figure 7A**).

Meta-analysis of 5 studies (Ren, 2005; Zhang et al., 2006; Yoshikawa et al., 2008; Zhu et al., 2012; Liu et al., 2018) showed a significant reduction of TUNEL-positive cells between the G-Rb1 group and control group (n = 67, SMD = -5.55, 95% CI -7.43 to -3.66, P < 0.01; Heterogeneity: Chi-square = 9.35, df = 4, P < 0.1, I² = 57%). By removing 1 study (Liu et al., 2018), which had a relatively high dosage (200 mg/kg), the pooled data was improved (n = 52, SMD = -4.74, 95% CI -5.97 to -3.51, P < 0.01; Heterogeneity: Chi-square = 1.47, df = 3, P > 0.1, I² = 0%) (**Figure 7B**).

Zhang et al. (2006) reported increased Bcl-x_l_ protein (P < 0.05) and mRNA levels (P < 0.01) in the ischemic cortex. And Fan, (2006), applying Nissl-staining, indicated a significant increase in Nissl-positive cells under G-Rb1 treatment.

Four other studies (Zhang et al., 1998; Zhang et al., 2006; Lu, 2010; Lu et al., 2011) using Nissl-staining showed a similar change without available data.

##### Anti-oxidation

**Figure 8:**
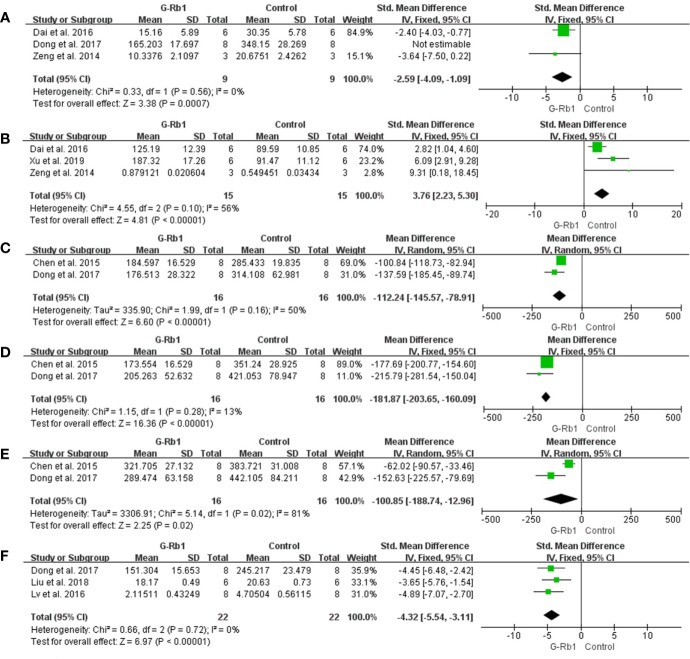
The pooled estimate of G-Rb1 on anti-oxidation according to: **(A)** MDA level; **(B)** SOD level; **(C)** NOX-1 mRNA; **(D)** NOX-4 mRNA; **(E)** NOX activity; **(F)** NO level.

Three studies (Zeng et al., 2014; Dai et al., 2016; Dong et al., 2017) based on measurements of MDA levels showed a significant difference between the G-Rb1 group and the control group (n = 34, SMD = -4.31, 95% CI -7.47 to -1.16, P < 0.01; Heterogeneity: Chi-square = 7.67, df = 2, P < 0.1, I² = 74%). After removal of 1 study (Dong et al., 2017), which studied the preventive effect of G-Rb1, the result stayed statistically homogeneous (n = 18, SMD = -2.59, 95% CI -4.09 to -1.09, P < 0.01; Heterogeneity: Chi-square = 0.33, df = 1, P > 0.1, I² = 0%) (**Figure 8A**).

Three studies reported an increase in SOD levels (n = 30, SMD = 3.76, 95% CI 2.23 to 5.30, P < 0.01; Heterogeneity: Chi-square = 4.55, df = 2, P = 0.1, I² = 56%) (**Figure 8B**).

Two studies (Chen et al., 2015; Dong et al., 2017) evaluated the difference in NOX expression and activity between the G-Rb1 and control groups. The pooled data showed an obvious reduction in the expression of NOX-1 mRNA (n = 32, MD = -112.24, 95% CI -145.57 to -78.91, P < 0.01; Heterogeneity: Chi-square = 1.99, df = 1, P > 0.1, I² = 50%) (**Figure 8C**), NOX-4 mRNA (n = 32, MD = -181.87, 95% CI -203.65 to -160.09, P < 0.01; Heterogeneity: Chi² = 1.15, df = 1, P > 0.1, I² = 13%) (**Figure 8D**) and NOX activity (n = 32, MD = -100.85, 95% CI -188.74 to -12.96, P < 0.05; Heterogeneity: Chi-square = 5.14, df = 1, P > 0.1, I² = 81%) (**Figure 8E**).

Three studies (Lv et al., 2016; Dong et al., 2017; Liu et al., 2018) assessed the NO level and meta-analysis indicated a significant effect of G-Rb1 (n = 44, SMD = -4.32, 95% CI -5.54 to -3.11, P < 0.01; Heterogeneity: Chi-square = 0.66, df = 2, P > 0.1, I² = 0%) (**Figure 8F**). Furthermore, Dong et al., (2017) reported increased GSH levels (n = 16, P < 0.01), while Lv, (2011) reported reduced ROS levels (n = 6, P < 0.05) when comparing the G-Rb1 group with the control group.

##### Anti-inflammation

**Figure 9:**
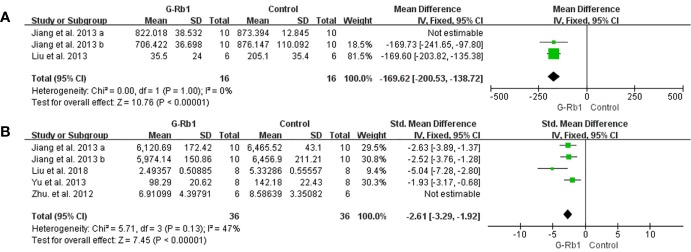
The pooled estimate of G-Rb1 on anti-inflammation according to: **(A)** IL-1 level; **(B)** TNF-α level.

Two studies with 3 comparisons (Jiang et al., 2013; Liu et al., 2013) reported a significant reduction of IL-1 levels but with substantial heterogeneity (n = 52, MD = -127.66, 95% CI -219.21 to -36.11, P < 0.01; Heterogeneity: Chi-square = 33.81, df = 2, P < 0.1, I² = 94%). Among these comparisons, Jiang et al. (2013) explored the preventive effect of G-Rb1 on the MCAO model, while others studied therapeutic effects. The pooled data changed to statistical homogeneity after removing the former study from the comparison (n = 32, MD = -169.62, 95% CI -200.53 to -138.72, P < 0.01; Heterogeneity: Chi-square = 0, df = 1, P > 0.1, I² = 0%) (**Figure 9A**).

Three studies with 5 comparisons (Zhu et al., 2012; Jiang et al., 2013; Yu et al., 2013; Liu et al., 2018) showed a significant reduction in TNF-α (n = 84, SMD = -2.31, 95% CI -3.53 to -1.09, P < 0.01; Heterogeneity: Chi-square = 16.23, df = 4, P < 0.1, I² = 75%). Removing 1 study (Zhu et al., 2012) led to an improved result (n = 72, SMD = -2.61, 95% CI -3.29 to -1.92, P < 0.01; Heterogeneity: Chi-square = 5.71, df = 3, P > 0.1, I² = 47%) (**Figure 9B**).

Liu et al. (2018) (P < 0.01) and Yu et al. (2013) (P < 0.01) indicated an obvious reduction in IL-6 when treated with G-Rb1 while Zhu et al. (2012) reported no statistical significance. Increased HMGB1 (P < 0.01) was reported by Liu et al., 2018. Four studies (Zhu et al., 2012; Zeng et al., 2014; Lv et al., 2016; Liu et al., 2018) indicated that G-Rb1 can significantly regulate the activity of the NF-κB signaling pathway by inhibiting the expression of relative proteins (P < 0.05).

##### Energy Metabolism and Cerebral Blood Flow

**Figure 10:**
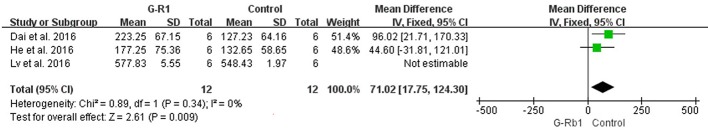
The pooled estimate of G-Rb1 on improving cerebral blood flow.

Li et al. (2005) (n = 10, P < 0.05) and Tao (Tao, 2004) (n = 9, P < 0.05) showed increased protein and mRNA levels of GLUT 1 and GLUT 3. Three studies (Dai et al., 2016; He et al., 2016; Lv et al., 2016) investigated the change of cerebral cortex blood flow following MCAO. After the removal of 1 study (Lv et al., 2016) which used a different animal species (C57BL/6J mice) with a relatively shorter occlusion time (1 h), meta-analysis showed a significant improvement in the G-Rb1 group (n = 24, MD = 71.02 95% CI 17.75 to 124.30, P < 0.01; Heterogeneity: Chi² = 0.89, df = 1, P > 0.1, I² = 0%) (**Figure 10**).

## Discussion

### Summary of Results

This is the first preclinical systematic review to evaluate the efficacy of G-Rb1 for experimental cerebral ischemia/reperfusion injury. Twenty-eight studies with 937 animals were selected. The quality of the included studies was generally moderate. The evidence available from the present study showed that G-Rb1 substantially reduced IV and improved NFS and BWC in focal cerebral ischemia animal models. Thus, G-Rb1 exerted a potential neuroprotective function, largely through the promotion of neurogenesis; anti-apoptosis, anti-oxidative and anti-inflammatory effects; and the improvement of energy metabolism and cerebral circulation.

### Limitations

Clinically, ischemic stroke usually occurs in elderly patients or those with hypertension or diabetes. However, only one study structured an experimental ischemic stroke model in aged rats and two studies in hypertensive rats. Several other methodological weaknesses also existed in the primary studies. Nineteen (76%) studies claimed randomization, but only 4 (16%) trials provided specific information as to the randomization generation. Blinded assessment of outcomes is necessary to minimize performance and detection bias, but only 9 (36%) studies mentioned a masked assessment of outcomes. An adequate sample size is important for study design, but only one study described a sample size calculation. Lacking a formal sample size calculation results in dubious statistical analysis validity.

### Implications

Ginseng is a famous herbal medicine and has been deeply researched. Ginsenosides, the major active pharmacological constituents of ginseng, are usually divided into the 20 (S)-protopanaxatriol group (ginsenosides Re, Rf, Rg1, Rg2, and Rh1) and the 20 (S)-protopanaxadiol group (ginsenosides Rb1, Rb2, Rc, Rd, Rg3 and Rh2) (Ong et al., 2015; Jin et al., 2019). G-Rb1 is considered to be the major ginsenoside (Kim, 2018), which is enriched in the roots and is also present in stems and leaves (Yu et al., 2019). A recent study illustrated that in the human body, G-Rb1 shows a longer half-life and a higher plasma concentration, compared to other ginsenosides, when taking red ginseng extract for 2 weeks, suggesting a stable absorption and slow elimination process (Jin et al., 2019). In terms of non-clinical pharmacokinetic behavior, the bioavailability of Rb1 in rats is about 1.18–4.35% with slowed renal excretion when the oral dose is about 10-104 mg/kg. (Won et al., 2019).

G-Rb1 has been proven to be linked to a wide range of biological activities, such as its neuroprotective role and antitumor activities. In the present study, we reviewed the possible neuroprotective mechanisms of G-Rb1 on experimental ischemic stroke and summarized as follows:

- **Reduction of brain edema** Brain edema is significantly associated with the poorer functional outcomes of ischemic stroke. Aquaporin-4 is a significant water channel protein mainly expressed in astrocytes throughout the central nervous system (CNS), especially in foot processes at the blood-brain barrier (BBB) (Verkman et al., 2017). A recent study showed that G-Rb1 can significantly reduce the content of AQP4 in the ischemic penumbra, hippocampus, and striatum (Li et al., 2018), and reduce brain edema. Additionally, matrix metalloproteinases have been shown to be strongly associated with brain edema after BBB disruption (Chen et al., 2015), while cerebral vessel formation and stabilization can alleviate brain edema (Lu, 2010), which is important for the development of new therapeutic approaches.
- **Promotion of neurogenesis** G-Rb1 had a positive effect on neurogenesis probably through increasing BDNF and Gap-43 levels (Jiang et al., 2013; Gao, 2017). BDNF is highly expressed in the CNS and contributes to the maintenance of neurons. BDNF/TrkB system and its downstream intracellular signaling pathways, such as ERK-, Akt-, and PLCγ-pathways, are necessary conditions for neuron survival and synaptic plasticity (Numakawa et al., 2017). Axonal growth cones guide and promote the growth of axons during nervous system development and regeneration in areas where GAP-43 is abundantly expressed (Kusik et al., 2010). In addition, G-Rb1 promotes the growth of neurons and axonal branches probably through activating the cAMP-PKA-CREB signaling pathway (Gao, 2017) or inhibiting the NgR/PhoA signaling pathway (Ren, 2005).
- **Anti-apoptosis** Apoptosis is an essential part of the pathogenesis of acute and/or chronic neurodegenerative diseases, for example, ischemic stroke, which can be responsible for neuronal death and irreversible cerebral dysfunction (Khoshnam et al., 2017). Inhibition of apoptosis could alleviate cerebral injury in stroke models (Radak et al., 2017). Caspase is the general name of the cysteine proteases family, which dominates the apoptosis process. Caspase-2, -8, -9, -10, -11, and -12 are regarded as initiator Caspases that are closely related to pro-apoptotic signals. Once initiator Caspases are activated, downstream effector Caspases, such as Caspase-3, begin to cleave targeted cellular proteins and perform apoptosis (Kuranaga and Miura, 2007). G-Rb1 was reported as contributing to the reduction of Caspase-3 (Fan, 2006; Gao et al., 2010; Zeng et al., 2014; Lv et al., 2016) and causing an anti-apoptosis effect.
- **Anti-oxidative activity** Oxidative stress plays an important role in the pathogenesis of ischemic stroke (Luo et al., 2018). Free radicals can be excessively produced–particularly in the ischemic areas–promoting lipid peroxidation, protein breakdown and DNA damage, which leads to cellular apoptotic neuronal damage (Milanizadeh et al., 2018). G-Rb1 plays an antioxidative role through increasing the activity of SOD (Zeng et al., 2014; Dai et al., 2016) and GSH levels (Dong et al., 2017) and decreasing the concentration of MDA (Zeng et al., 2014; Dai et al., 2016; Dong et al., 2017), NO (Lv et al., 2016; Dong et al., 2017; Liu et al., 2018) and the activity and expression of NOX (Chen et al., 2015; Dong et al., 2017). The pooled data indicated that G-Rb1 significantly inhibited oxidative stress reactions and reduced the neurotoxicity of free radicals.
- **Anti-inflammation** The present study showed that G-Rb1 exerts anti-inflammatory effects by decreasing the expression of pro-inflammatory cytokine IL-1 (Jiang et al., 2013; Liu et al., 2013; Chen et al., 2015), IL-6 (Zhu et al., 2012; Yu et al., 2013; Liu et al., 2018) and TNF-α (Zhu et al., 2012; Jiang et al., 2013; Yu et al., 2013; Liu et al., 2018) as well as the expression of HMGB1 (Liu et al., 2018). In addition, the NF-κB signaling pathway (Zhu et al., 2012; Zeng et al., 2014; Lv et al., 2016; Liu et al., 2018) could be the mechanism through which this occurs. Inflammation is a well-recognized pathological event which can be responsible for secondary brain tissue damage following ischemic stroke (Rajkovic et al., 2018). The pro-inflammatory cytokines induce nerve tissue damage in the ischemic area of the brain largely through down-regulation of microcirculation, enhancing the pro-thrombotic processes and releasing other neurotoxic cytokines (Cieslak et al., 2013). HMGB1 is a well-researched non-histone DNA-binding protein located in the nucleus. Once there are infections or tissue injuries, HMGB1 can be up-regulated by immune cells or necrotic cells (Lotze and Tracey, 2005). In cerebral ischemia/reperfusion processes, it triggers delayed inflammation and exacerbates neuron damage (Kim et al., 2018). On the other hand, the NF-κB family, consisting of transcription factors, and its associated signaling pathway play a complex but crucial role in the regulation of immune response (Tilborghs et al., 2017).
- **Improvement of energy metabolism** Neurons have higher energy expenditure and lower reserves compared to other cell types (Belanger et al., 2011). After cerebral ischemia a lack of glucose and oxygen, and consequently adenosine triphosphate, is one of the major events that results in energy failure (Hu et al., 2017). Numerous interactions of transporters, enzymes, and intracellular signaling processes within the neurovascular unit at the BBB participate in glucose transport, where GLUT1 and GLUT3 are regarded as the major glucose transporters (Patching, 2017). In the present study, G-Rb1 increased the expression of GLUT1 and GLUT3 in cerebral ischemic penumbra indicating that it can maintain the energy supply of the injured brain, which may be one of the mechanisms of its protective effect (Tao, 2004; Li et al., 2005).
- **Improvement of cerebral circulation** In cerebral ischemic stroke, clinical evidence shows that early revascularization is a critical process for rescuing salvageable tissue (Wardlaw et al., 2014; Goyal et al., 2016). In the present study, 3 studies that were investigated found that G-Rb1 has a positive effect on improving cerebral blood flow (Dai et al., 2016; He et al., 2016; Lv et al., 2016) probably by activating A2a receptors and the cAMP-PKA-KATP signaling pathway in vascular endothelial cells and vascular smooth muscle cells.

**Figure 11:**
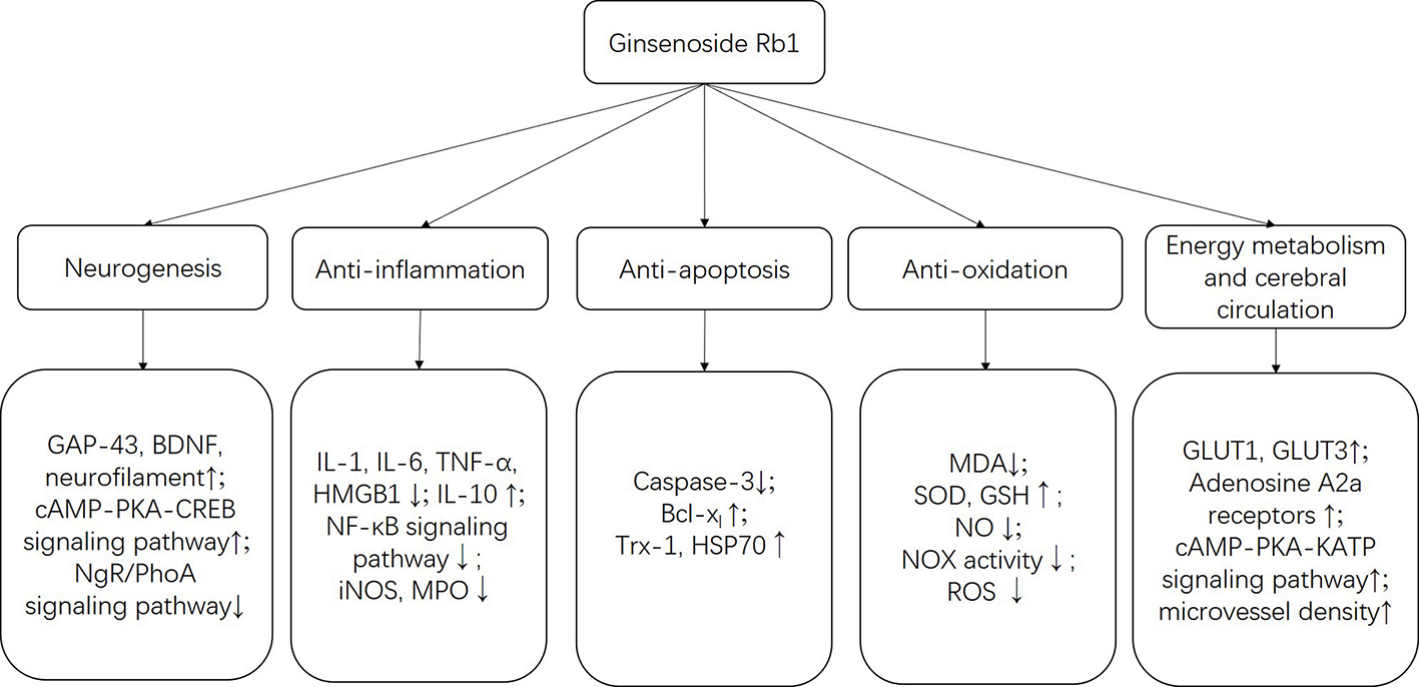
The schematic representation of neuroprotective mechanisms of G-Rb1 for ischemic stroke.

More details were summarized in **Table 4** and **Figure 11**. In addition to the mechanisms mentioned above, the specific molecular mechanism needs further research.

**Table 4:** Summary of mechanism studies of G-Rb1 on experimental ischemic stroke.

Study	Model	Method of administration (Experimental vs. Control)	Effects	Mechanisms
Chen et al., 2015	MCAO (t) 1h (ZL) in ICR mice	G-Rb1 vs. normal saline	↓ BWC; anti-inflammation; anti-oxidation; ↑ neurological function; ↓ cortical and hemispheric infarction.	↑ tight junction proteins occludin and ZO-1; ↓ MMP-9; ↓ pro-inﬂammatory factors iNOS and MPO activity; ↑anti-inﬂammatory marker arginase 1 and IL-10; ↓ expression of NOX1, NOX4, and NOX activity.
Dai et al., 2016	MCAO (t) 2h (ZL) in SD rats	G-Rb1 vs. normal saline	↑ cerebral blood flow; anti-oxidation; ↑ neurological function; ↓ brain infarction.	↑ adenosine, activating A2a receptors and cAMP-PKA-KATP signaling pathway in vascular smooth muscle cells; ↓ MDA and ↑ SOD expression.
Dong et al., 2017	MCAO (t) 1h (ZL) in C57BL/6J mice	G-Rb1 vs. normal saline	anti-oxidation; ↑ neurological function; ↓ brain infarction; ↓ brain water content.	↑ GSH, ↓ MDA, NO, NOX-1, NOX-4 expression and ↓ NOX activity; ↓ ERK1/2 signaling pathway.
Fan, 2006	MCAO (t) 2h (ZL) in SD rats	G-Rb1 vs. normal saline	anti-apoptosis; ↓ neuron death in ischemic penumbra; ↑ neurological function; ↓ brain infarction.	↓ Caspase-3 expression.
Gao, 2017	MCAO (p) in C57BL/6 mice	G-Rb1 vs. normal saline	↑ neurogenesis; ↑ BDA-labeled neurons and axonal branches; ↑ neurological function.	↑ GAP-43 expression; activating cAMP-PKA-CREB signaling pathway.
Gao et al., 2010	MCAO (t) 2h (ZL) in Wistar rats	G-Rb1 vs. normal saline	↑ neurogenesis, ↑ Nestin-positive cells; anti-apoptosis; ↑ neurological function.	↑ BDNF expression; ↓ Caspase-3 expression.
He et al., 2016	MCAO (t) 2h (ZL) in SD rats	G-Rb1 vs. normal saline	↑ cerebral blood flow; ↑ neurological function; ↓ brain infarction.	↑ adenosine, activating A2a receptors and cAMP-PKA-KATP signaling pathway in vascular endothelial cells.
Jiang et al., 2013	MCAO (t) 3h (ZL) in SD rats	G-Rb1 vs. no treatment	anti-inflammation; ↑ neurogenesis; ↓ brain infarction.	↓ IL-1 and TNF-α expression; ↑ BDNF, Gap-43 and neurofilament expression.
Li et al., 2005	MCAO (t) 1h (ZL) in SD rats	G-Rb1 vs. normal saline	↑ energy supplement; ↓ brain infarction.	↑ GLUT 3 expression.
Li et al., 2018	MCAO (t) 2h (ZL) in SD rats	G-Rb1 vs. normal saline	↓ BWC; ↑ neurological function; ↓ brain infarction.	↑ AQP4 and gap junctions Cx43 expression.
Liu et al., 2018	MCAO (t) 2h (ZL) in Wistar rats	G-Rb1 vs. normal saline	anti-inflammation; anti-oxidation; anti-apoptosis; ↑ neurological function; ↓ brain infarction.	↓ IL-6, TNF-α and iNOS expression; ↓ HMGB1 and NF-κB p65 expression; ↓ NO level.
Liu et al., 2013	MCAO (t) 2h (ZL) in SD rats	G-Rb1 vs. normal saline	anti-inflammation; ↑ neurological function; ↓ brain infarction.	↓ IL-1β expression.
Lu, 2010	MCAO (t) 1.5h (ZL) in SD rats	G-Rb1 vs. normal saline	anti-apoptosis, ↑ Nissl-positive cells; ↓ brain infarction.	↑ microvessel density in ischemic penumbra.
Lu et al., 2011	MCAO (t) 1.5h (ZL) in SD rats	G-Rb1 vs. normal saline	↓ autophagy activity; ↑ Nissl-positive cells; ↓ brain infarction.	↓ LC3II and Beclin 1 expression.
Lv, 2011	MCAO (t) 1h (ZL) in BALB/C mice	G-Rb1 vs. normal saline	anti-oxidation; ↑ neurological function; ↓ brain infarction.	↓ ROS level.
Lv et al., 2016	MCAO (t) 1h (ZL) in C57BL/6J mice	G-Rb1 vs. no treatment	↑ cerebral blood flow; anti-oxidation; anti-apoptosis; anti-inflammation; ↑ neurological function; ↓ brain infarction.	↓ NO level; ↓ Caspase-3 level; ↓ NF-κB signaling pathway.
Ren, 2005	MCAO (t) 2h (ZL) in SD rats	G-Rb1 vs. normal saline	↑ neurite growth; ↓ brain edema; anti-apoptosis, ↓ TUNEL-positive cells; ↑ neurological function; ↓ brain infarction.	↓ NgR/PhoA signaling pathway.
Tao, 2004	MCAO (t) 1h (ZL) in SD rats	G-Rb1 vs. normal saline	↑ energy supplement; ↑ neurological function; ↓ brain infarction.	↑ GLUTl and GLUT 3 expression.
Yoshikawa et al., 2008	MCAO (p) in Cynomolgus monkeys	G-Rb1 vs. normal saline	anti-apoptosis and ↓neuron damage, ↑ NeuN-positive cells and ↓ TUNEL-positive cells.	NR
Yu et al., 2013	MCAO (t) 2h (ZL) in SD rats	G-Rb1 vs. normal saline	anti-inflammation; ↑ neurological function, neuroprotection.	↓ TNF-α and IL-6 levels; ↓ JAK2/STAT3 signaling pathway.
Zeng et al., 2014	MCAO (t) 2h (ZL) in C57BL/6 mice	G-Rb1 vs. normal saline	anti-oxidation; anti-apoptosis; ↑ neurological function; ↓ brain infarction.	↓ MDA, ↑ SOD-1 expression; ↑Trx-1 and HSP70; ↓ Caspase-3 expression; restoring Akt/NF-κB signaling pathway.
Zhang et al., 2006	MCAO (p) in SHR-SP rats	G-Rb1 vs. normal saline	anti-apoptosis; ↓ TUNEL-positive cells; ↑ Nissl-positive cells; ↑ neurological function; ↓ brain infarction.	↑ Bcl-xl level.
Zhang et al., 1998	MCAO (p) in SHR-SP rats	G-Rb1 vs. normal saline	anti-apoptosis, ↑ Nissl-positive cells; ↑ neurological function.	NR
Zhu et al., 2012	MCAO (t) 2h (ZL) in SD rats	G-Rb1 vs. normal saline	anti-inflammation; anti-apoptosis; ↓ TUNEL-positive cells.	↓ TNF-α and IL-6 levels; ↓ NF-κB signaling pathway.
Sun and Xu, 2018	MCAO (t) 2h (ZL) in SD rats	G-Rb1 vs. normal saline	↑ neurological function.	↑PI3K/AKT signaling pathway.
Shi et al., 2019	MCAO (t) 0.5h (ZL) in SD rats	G-Rb1 vs. no treatment	↑ neurological function; ↓ brain edema; anti-oxidation.	↑ Cx40 ↓ NOX activity
Xu et al., 2019	MCAO (t) 2h (ZL) in SD rats	G-Rb1 vs. normal saline	↑ neurological function; ↓ brain infarction; anti-oxidation.	↓ SOD

SD, Sprague—Dawley; SHR-SP, Spontaneously Hypertensive Rats-Stroke Prone; ZL, Zea Longa; MCAO, middle cerebral artery occlusion; h, hour; G-Rb1, Ginsenoside-Rb1; t, temporary; p, permanent; BWC, brain water content; BDNF, brain derived neurotrophic factor; HMGB1, high-mobility group box 1; MDA, malondialdehyde; SOD, superoxide dismutase; GSH, glutathione; ROS, reactive oxygen species; NO, nitric oxide; NOX, nicotinamide adenine dinucleotide phosphate oxidase; MMP-9, matrix metalloproteinase-9; MPO, myeloperoxidase; GLUT, glucose transporters; NR, not reported; ↑, upregulation; ↓, downregulation.

In the present study, chloral hydrate is the most common anesthetic used in 16 (64%) included studies. Although it has relatively few effects on cardiovascular function and no neuroprotection in stroke studies (Ozden and Isenmann, 2004), chloral hydrate has raised questioned regarding its prolonged recovery, mutagenic effects, and carcinogenic effects in animal use. Currently, isoflurane has been suggested as a replacement for chloral hydrate, because it is easy to administer and to titrate, has a rapid onset and recovery period, an adequate and reproducible anesthesia depth, minimal cardiac depression, and ethical considerations (Maud et al., 2014). Although previous studies demonstrated that anesthetics including ketamine, isoflurane, and halothane had potential neuroprotective effects (Kapinya et al., 2002; Xiong et al., 2003; Kitano et al., 2007b; Wang et al., 2008; Deng et al., 2014; Tang et al., 2015), isoflurane can be an appropriate anesthetic agent when establishing the MCAO model (Kitano et al., 2007a).

Ischemic stroke animal models are an indispensable tool for identifying the mechanisms of ischemic stroke and developing new agents for stroke therapy. Currently, stroke experiments are mainly carried out in small animals, such as mice, rats and rabbits (Fluri et al., 2015). Rodents are one of the most commonly used animals in stroke studies for the following advantages: the cerebrovascular system and physiology of the rat is similar to that of humans (Yamori et al., 1976); its moderate body size allows easy monitoring of physiological parameters; its small brain size is makes it ideal for fixed procedures, for example, *in vivo* cryocapture for biochemical analysis (Ponten et al., 1973); there is a relative homogeneity within strains (Strom et al., 2013); and most of all, it is easy to conduct reproducible studies. In addition, large animal models, such as non-human primates, are more similar to human anatomy and pharmacodynamics (van Hout et al., 2016); however, it has weaknesses as they are more expensive, difficult to manipulate, and raise accompanying ethical issues. Thus, in future research, we should select an ideal model that usually is a biological representative of human disease, inexpensive, reproducible, easily manipulated, and ethically sound according to the experimental purpose.

There are some differences between animal and human ischemic strokes. For one thing, the anatomy of the brain and cerebral vessels varies between species, which results in different patterns of ischemic damage (Sommer, 2017). For another, ischemic stroke in humans preferentially affects elderly patients with multiple risk factors of cerebrovascular diseases (such as diabetes mellitus, hypertension, hyperlipidemia, obesity), suggesting that the heterogenicity of human stroke requires complex interventions (Fluri et al., 2015). Experimental stroke is usually performed on young, healthy, male rodents and under highly standardized and well-controlled conditions, which might be beneficial for reducing infarct volume (Dirnagl, 2016; Becker, 2016). In addition, anesthesia can be another influence factor. It is reported that anesthetics play a role in affecting blood pressure, CBF and metabolism. Moreover, anesthetics may have neuroprotective effects, thus modulating some aspects of post-ischemic processes (Koerner and Brambrink, 2006).

Blood hypercoagulability and thromboembolism play important roles in the pathophysiology of cerebrovascular disease. A recent study evaluated a new-developed “nanoplatelet” (tP-NP-rtPA/ZL006e) in rats that can deliver thrombolytics and neuroprotectants sequentially to the site of the thrombus and ischemic penumbra, for a maximized combinational effect (Xu et al., 2019). Meanwhile, G-Rb1 was found to have anticoagulant and antianemia effects in rodents with cancer (Zheng et al., 2019), which could be a potential neuroprotective agent from the perspective of pathophysiology.

Converting experimental results into clinically effective treatment methods creates a bottleneck, especially in the research of cerebrovascular diseases. Thus, preclinical research process must be rigorously designed and closely follow the preset protocols, which could strengthen the internal validity of experimental studies (Zinsmeister and Connor, 2008). Stroke Therapy Academic Industry Roundtable has given suggestions to improve the quality of preclinical studies and improve the rate of clinical translation (STAIR, 1999). As for preclinical systematic reviews, according to Moher et al., there would be around a 40% magnification of treatment efficacy statistically when low-quality trials are included in the pool (Moher et al., 1998). Thus, we recommend classic CAMARADES 10-item checklist (Macleod et al., 2004), which is an international collaboration established in 2004, aiming to support meta-analyses of animal data, especially for experimental ischemic stroke.

## Conclusion

Pooled data from the present study demonstrated that G-Rb1 can reduce IV, attenuate NFS and BWC, and thus have potential neuroprotective effects in the animal model of ischemic stroke, largely through enhancing neurogenesis, anti-apoptosis, anti-oxidation, anti-inflammation, improving energy metabolism and improving cerebral circulation. Although some factors such as study quality and methodological flaws may undermine the validity of positive findings, this systematic review provides an experimental evidence-based approach to translate new therapies for ischemic stroke.

## Data Availability Statement

All datasets generated for this study are included in the article/**Supplementary Material**.

## Author Contributions

Y-HS and YL contributed to the conception and design of the study. Y-HS, YL, YW, ZX, and HF contributed to study selection, data extraction, analysis, and/or interpretation. G-QZ provided the final approval and takes overall responsibility for this published work.

## Funding

This study was supported by a grant of Young and Middle-Aged University Discipline Leaders of Zhejiang Province, China [2013277]; Zhejiang Provincial Program for the Cultivation of High-level Health talents [2015].

## Conflict of Interest

The authors declare that the research was conducted in the absence of any commercial or financial relationships that could be construed as a potential conflict of interest.

## Supplementary Material

Supplementary Image 1 Subgroup analysis of G-Rb1 on improving infarct volume according to different doses.

Supplementary Image 2 Subgroup analysis of G-Rb1 on improving infarct volume according to different treatment times.

Supplementary Table 1 PRISMA 2009 Checklist.

## Abbreviations

CBF, cerebral blood flow; tPA, tissue-type plasminogen activator; G-Rb1, Ginsenoside-Rb1; MCAO, middle cerebral artery occlusion; NFS, neurological function score; IV: infarct volume; BBB, blood-brain barrier; BWC, brain water content; MD, mean difference; CI, confidence interval; SMD, standardized mean difference; SD rats, Sprague-Dawley rats; SHRSP, stroke-prone spontaneously hypertensive rats; ICR, Institute of Cancer Research; ZL, Zea longa; mNSS, modified neurological severity score; TTC, triphenyl tetrazolium chloride; HE, hematoxylin-eosin; BDNF, brain-derived neurotrophic factor; HMGB1, high-mobility group box 1; MDA, malondialdehyde; SOD, superoxide dismutase; GSH, glutathione; ROS, reactive oxygen species; NO, nitric oxide; NOX, nicotinamide adenine dinucleotide phosphate oxidase; GLUT, glucose transporters; CNS, central nervous system; CAMARADES, Collaborative Approach to Meta-Analysis and Review of Animal Data in Experimental Studies; h, hours; d, days.
