# Supplementary figures and images for: Ginsenoside-Rb1 for Ischemic Stroke: A Systematic Review and Meta-analysis of Preclinical Evidence and Possible Mechanisms
Source: Front Pharmacol. 2020 Mar 31;11:285. doi: 10.3389/fphar.2020.00285 (PMC7137731; doi:10.3389/fphar.2020.00285)

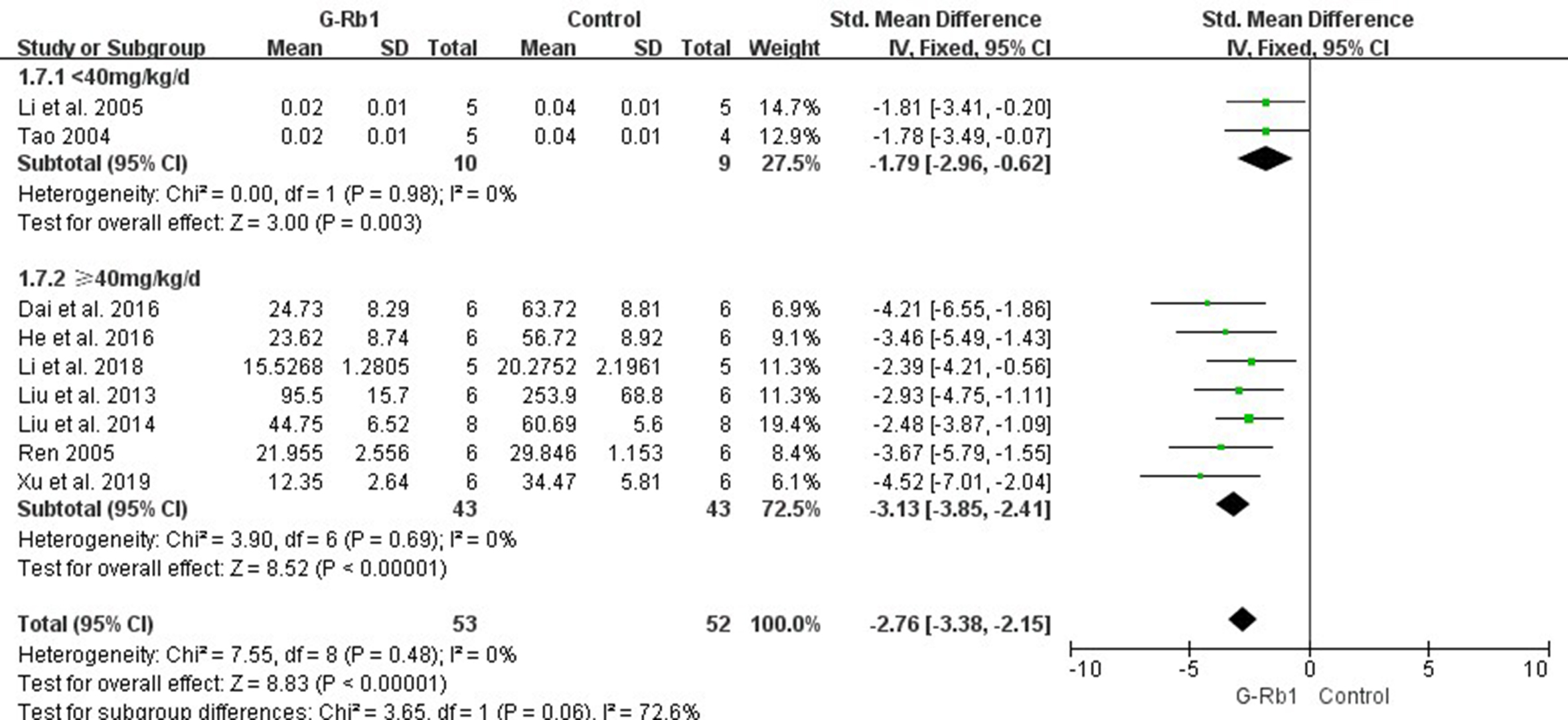

Supplement: Supplementary Image 1 — Subgroup analysis of G-Rb1 on improving infarct volume according to different doses. [file Image_1.jpeg]

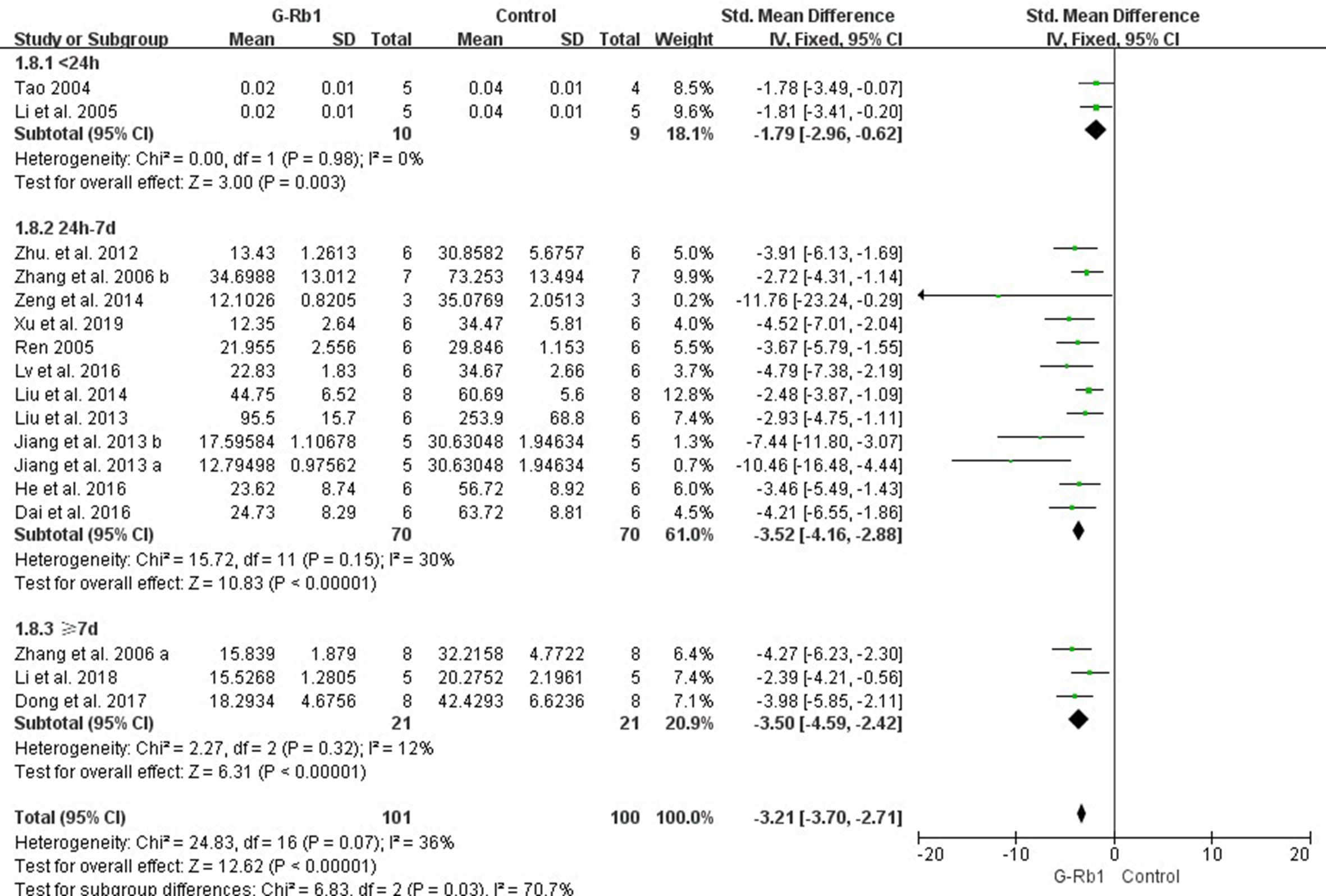

Supplement: Supplementary Image 2 — Subgroup analysis of G-Rb1 on improving infarct volume according to different treatment times. [file Image_2.jpeg]
